# Sialidase inhibitors attenuate pulmonary fibrosis in a mouse model

**DOI:** 10.1038/s41598-017-15198-8

**Published:** 2017-11-08

**Authors:** Tejas R. Karhadkar, Darrell Pilling, Nehemiah Cox, Richard H. Gomer

**Affiliations:** Department of Biology, Texas A&M University, 301 Old Main Drive, College Station, Texas 77843-3474 USA

## Abstract

Fibrosis involves increasing amounts of scar tissue appearing in a tissue, but what drives this is unclear. In fibrotic lesions in human and mouse lungs, we found extensive desialylation of glycoconjugates, and upregulation of sialidases. The fibrosis-associated cytokine TGF-β1 upregulates sialidases in human airway epithelium cells, lung fibroblasts, and immune system cells. Conversely, addition of sialidases to human peripheral blood mononuclear cells induces accumulation of extracellular TGF-β1, forming what appears to be a sialidase - TGF-β1 - sialidase positive feedback loop. Monocyte-derived cells called fibrocytes also activate fibroblasts, and we found that sialidases potentiate fibrocyte differentiation. A sialylated glycoprotein called serum amyloid P (SAP) inhibits fibrocyte differentiation, and sialidases attenuate SAP function. Injections of the sialidase inhibitors DANA and oseltamivir (Tamiflu) starting either 1 day or 10 days after bleomycin strongly attenuate pulmonary fibrosis in the mouse bleomycin model, and by breaking the feedback loop, cause a downregulation of sialidase and TGF-β1 accumulation. Together, these results suggest that a positive feedback loop involving sialidases potentiates fibrosis, and suggest that sialidase inhibitors could be useful for the treatment of fibrosis.

## Introduction

Fibrosing diseases such as severe asthma, ischemic heart disease, cirrhosis of the liver, end stage kidney disease, and idiopathic pulmonary fibrosis (IPF) involve the inappropriate formation of scar tissue in an internal organ, and are associated with an estimated 45% of all deaths in the US^1–4^. In these diseases, insults to the tissue, such as particulate matter or toxins in the lungs, initiate an inappropriate and unnecessary wound healing response, leading to organ failure and death^3–6^. What drives the fibrosis is poorly understood.

Many secreted and cell-surface mammalian proteins are glycosylated, and many of the glycosylation structures have sialic acids as the monosaccharide at the distal tip or tips of the polysaccharide on the protein^7–9^. Some viruses, bacteria, protozoa, and all mammals have sialidases (also known as neuraminidases) that remove the sialic acids from glycoconjugates^10,11^. Viruses such as influenza require sialidase to release the virus from the sialic acids on the outside of a host cell, and the sialidase inhibitors oseltamivir (Tamiflu) and zanamivir (Relenza) are front-line therapeutics for influenza^12^. The bacterial respiratory pathogen *Pseudomonas aeruginosa* uses a sialidase to colonize the lungs^13^. Mammals have four sialidases, NEU1 – NEU4. NEU1, 2, and 4 prefer α-(2,3) linked sialic acids as a substrate, while NEU3 prefers α-(2,6)^10,14,15^. NEU1 is in the lysosome^16–18^, NEU2 is a soluble, cytosolic enzyme, and NEU4 has 2 isoforms, one on mitochondria, and the other on intracellular membranes^15,19,20^. NEU3 is in endosomes and the extracellular side of the plasma membrane, and under some conditions can be released from the membrane to the extracellular environment^21^.

The serum glycoprotein Serum Amyloid P (which has an α-(2,6)-linked terminal sialic acid) appears to have a calming effect on the innate immune system, and inhibits fibrosis in animal models and in early-stage clinical trials^22–29^. C-reactive protein (CRP) is closely related to SAP, but is not glycosylated^30^. Unlike SAP, CRP generally potentiates inflammation and fibrosis^31^. We mutated SAP protein surface amino acids that were different from CRP, and could not find a domain on the SAP protein surface that when mutated strongly altered SAP function^32,33^. However, when SAP was desialylated with sialidase, the effects of SAP were largely abrogated^34^. When CRP was mutated to have a glycosylation similar to that of SAP (including a terminal sialic acid), the resulting CRP A32N was essentially indistinguishable from SAP in *in vitro* assays on neutrophils, monocytes, and macrophages^34^. Together, these results indicated that a terminal sialic acid on SAP plays a key role in its ability to regulate the innate immune system.

Intravenous immunoglobulin therapy is a treatment for some autoimmune diseases, where the intravenous immunoglobulin seems to act as an immunosuppressant^35^. Immunoglobulins are glycosylated, and there is a heterogeneity in the extent to which the glycosylations have terminal sialic acids^36^. Fractionation of immunoglobulins, as well as treatment of immunoglobulins with sialidase, showed that only immunoglobulins with terminal sialic acids act as immunosuppressants^37,38^. These results support the hypothesis that a lack of glycoconjugates with sialic acids permits inflammation. A variety of studies indicate that sialidases potentiate inflammation^39–46^. Conversely, other studies indicate that inflammation potentiates sialidase activity, with most of the reports showing that NEU1 is associated with inflammation^43,47–52^.

In a study on patients with idiopathic pulmonary fibrosis (IPF), the bronchoalveolar lavage (BAL) fluid from 8 of 9 patients had a high sialidase activity, while the BAL fluid from 9 healthy controls showed no detectable sialidase activity^53^. In the 3 IPF patients where BAL was collected in subsequent years, the BAL fluid sialidase activity increased as the disease progressed. A recent report also found increased levels of NEU1 in lung fibroblasts of some but not all pulmonary fibrosis patients, and that adenoviral administration of NEU1 promoted bleomycin-induced lung inflammation and fibrosis^54^. In this report, we show that inhibiting sialidases can inhibit fibrosis in a mouse model.

## Results

### There is increased desialylation in fibrotic lungs

**Figure 1:**
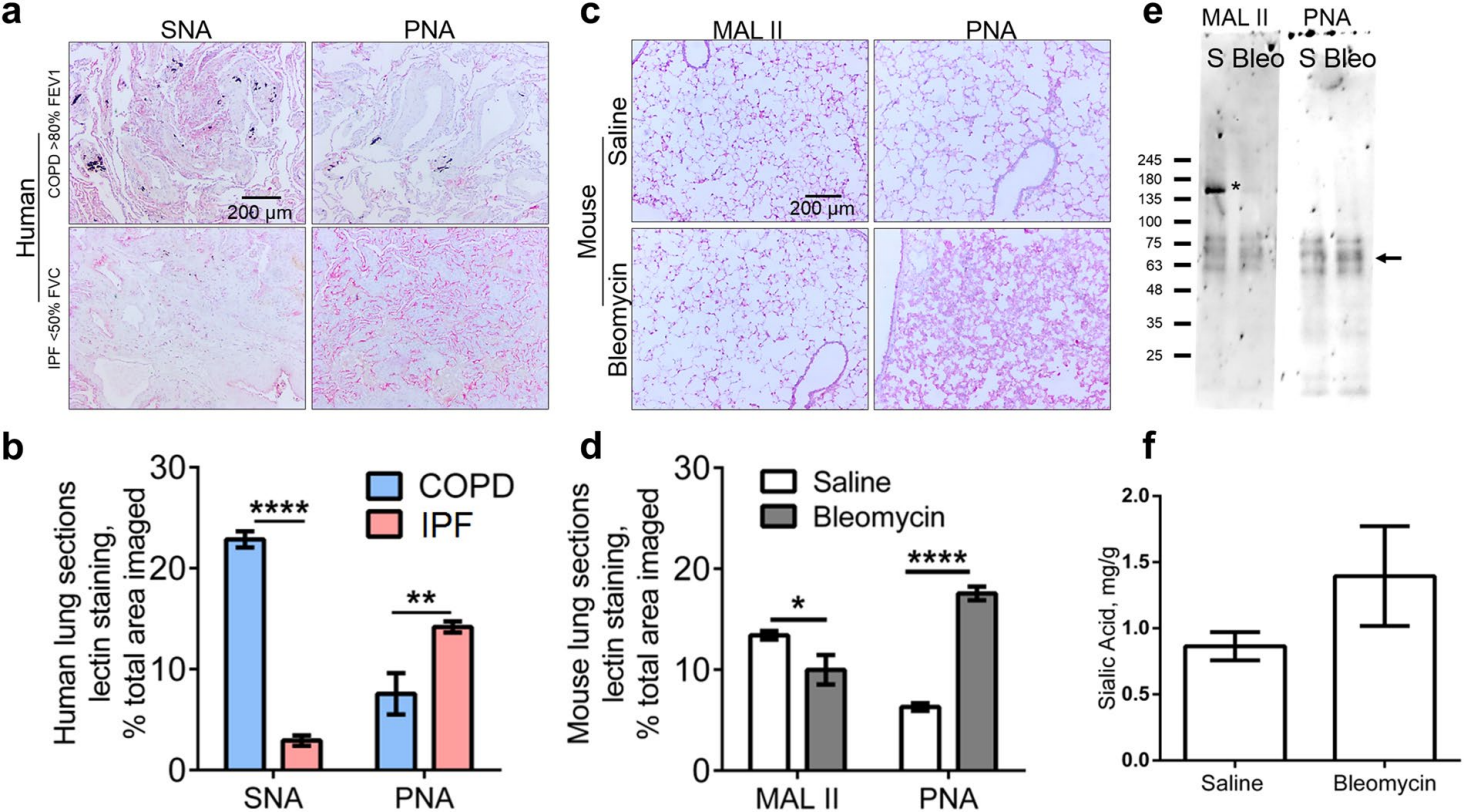
There is decreased sialylation in pulmonary fibrosis. (**a**) Human lung sections were stained with SNA to detect sialic acids, or PNA to detect desialylated polysaccharides. IPF < 50% FVC indicates a pulmonary fibrosis patient with poor lung function; COPD > 80% FEV1 indicates a chronic obstructive pulmonary disease patient with relatively normal lung function. Positive staining is red, with nuclei counterstained blue. Bar is 0.2 mm. (**b**) Quantification of lectin staining for human lung sections with ImageJ. (**c**) Mouse lungs at day 21 after saline or bleomycin treatment were stained with MAL II to detect sialic acid on glycoconjugates or with PNA to detect desialylated polysaccharides. Bar is 0.2 mm. (**d**) Quantification of lectin staining for mouse lung sections with ImageJ. For (B) and (D), values are mean ± SEM, n = 3 or 4; **indicates p < 0.005, ****p < 0.0001 (t-test). (**e**) BAL fluid from mice at day 21 after saline (S) or bleomycin were analyzed by western blotting, staining with MAL II or PNA. *Indicates bleomycin causing decreased sialylation on a protein and arrow indicates bleomycin causing the gain of a desialylated protein. Molecular masses in kDa are at left. Images in A, C, and E are representative of 4 patients or 3 mice per group. (**f**) Total sialic acid from the day 21 lung tissue of saline- or bleomycin-treated mice. Values are mean ± SEM, n = 3.

To determine if desialylation of proteins can be observed in pulmonary fibrosis, we stained tissue sections with *Sambucus nigra* lectin (SNA) or *Maackia amurensis* lectin II (Mal II), which detect sialic acids on glycoconjugates, or peanut agglutinin (PNA), which detects a variety of carbohydrates if they are not sialylated^46^. Compared to chronic obstructive pulmonary disease (COPD) patient lungs with relatively normal lung function (see Supplementary Table S1 for patient details), or compared to control mouse lungs (intratracheal saline instead of intratracheal bleomycin), fibrotic human lungs and fibrotic mouse lungs showed less staining for sialylated glycoconjugates, and increased staining for desialylated glycoconjugates (Fig. 1a–d). In the bronchoalveolar lavage (BAL) fluid at day 21 from bleomycin-treated mice, we observed increased amounts of a desialylated protein and a decreased amount of a sialylated protein (Fig. 1e).

Sialyltransferases catalyze the sialylation of glycoconjugates. There are 20 identified mammalian sialyltransferases^55,56^. Three representative sialyltransferases are ST3GAL2 and ST6GAL2, which sialylate oligosaccharides, and ST8SIA1, which sialylates gangliosides^55,57^. In bleomycin-induced fibrotic lesions in mouse lungs, we observed increased levels of ST3GAL2 and ST6GAL2, and no change in levels of ST8SIA1 (Supplementary Fig. S1), suggesting that the reduced sialylation observed in Fig. 1a–e is not due to reduced levels of these 3 sialyltransferases. Fibrotic mouse lungs showed normal levels of total (free + conjugated) sialic acid (Fig. 1f), indicating that the reduced amount of sialic acid in glycoconjugates is not due to a reduced production of sialic acid.

### Sialidases are upregulated in fibrotic lungs

In addition to the observation that there is sialidase activity in the BAL fluid from patients with IPF^53^, the above results suggest the possibility that there may be increased sialidase protein in fibrotic lungs. To examine this, we stained lung sections from IPF patients and COPD patients with antibodies against the four human sialidases. The antibodies were made against domains of the sialidases that are different from each other, do not bind the other sialidases (Supplementary Fig. S2), and stain bands on western blots of whole cell lysates corresponding to bands observed by others (Supplementary Fig. S3)^42,54,58,59^.

**Figure 2:**
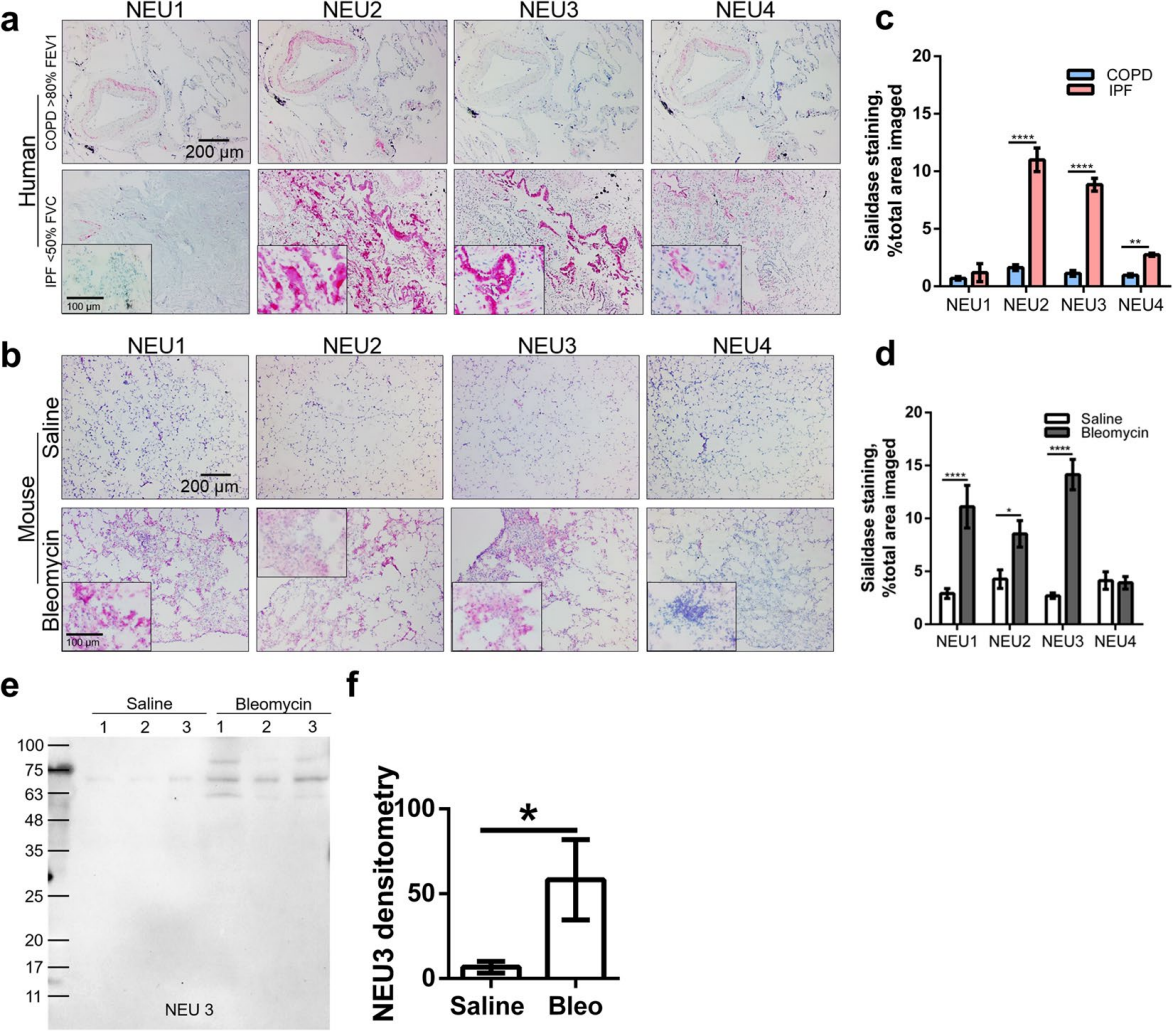
Some sialidases are upregulated in lung fibrosis. Human (**a**) and mouse (**b**) lung sections as in Fig. 1 were stained for the indicated sialidases. Outside image bars are at 0.2 mm. Inset in a and b are higher magnification images of the ILD IPF (**a**) or bleomycin (**b**) lungs; bars are 0.1 mm. All images are representative of 4 patients or 3 mice per group. Quantification of sialidase staining for human (**c**) and mouse (**d**) lung sections with ImageJ. Values are mean ± SEM, n = 3 or 4; *indicates p < 0.05, ****p < 0.0001 (t-test). (**e**) BAL fluid from mice at day 21 after saline or bleomycin were stained for sialidase3. 1, 2, and 3 refer to different individual mice. Positive staining appears black. Molecular masses in kDa are at left. (**f**) Quantification of NEU3 (**e**) western blot, values expressed in percent relative density of black bands. Values are mean ± SEM, n = 3 or 4; *indicates p < 0.05 (t-test).

The COPD lungs showed low levels of the four human sialidases (Fig. 2a). Two of three IPF patient lungs showed no significant staining for sialidase 1 (NEU1) while one IPF patient did have increased levels of NEU1 staining (Supplementary Fig. S4). Compared to the COPD patients, the ILD patient lungs had increased levels of NEU2, NEU3, and NEU4 (Fig. 2a and c). To confirm the specificity of the sialidase antibodies, we pre-incubated the NEU2 and NEU3 antibodies with recombinant NEU2 or NEU3 respectively, and found that this pre-treatment abrogated staining of fibrotic human lung tissue (Supplementary Fig S5). Mice treated with oropharyngeal bleomycin develop fibrotic lesions in the lungs at day 21^60^. Mice treated with oropharyngeal saline as a control had low levels of all four mouse sialidases in their lungs (Fig. 2b). Compared to the controls, bleomycin-treated mice had fibrotic lesions and increased levels of NEU1, NEU2, and NEU3, but no increase in NEU4 (Fig. 2b and d). Higher magnification images showed patchy distributions of the upregulated sialidases in fibrotic lesions (inset in Fig. 2a and b). In the BAL fluid from mice with bleomycin-induced lung fibrosis, NEU1, 2, and 4 were not detected, even on over-exposed western blots (Supplementary Fig. S6 a,b and c), while NEU3 was upregulated compared to the BAL fluid from control mice (Fig. 2e and f). For unknown reasons, one bleomycin-treated mouse also showed anti-NEU3 staining of a band with an apparent molecular mass higher than NEU3 (Fig. 2e) Together, these data indicate that the levels of some sialidases are increased in lung fibrosis in humans and mice.

**Figure 3:**
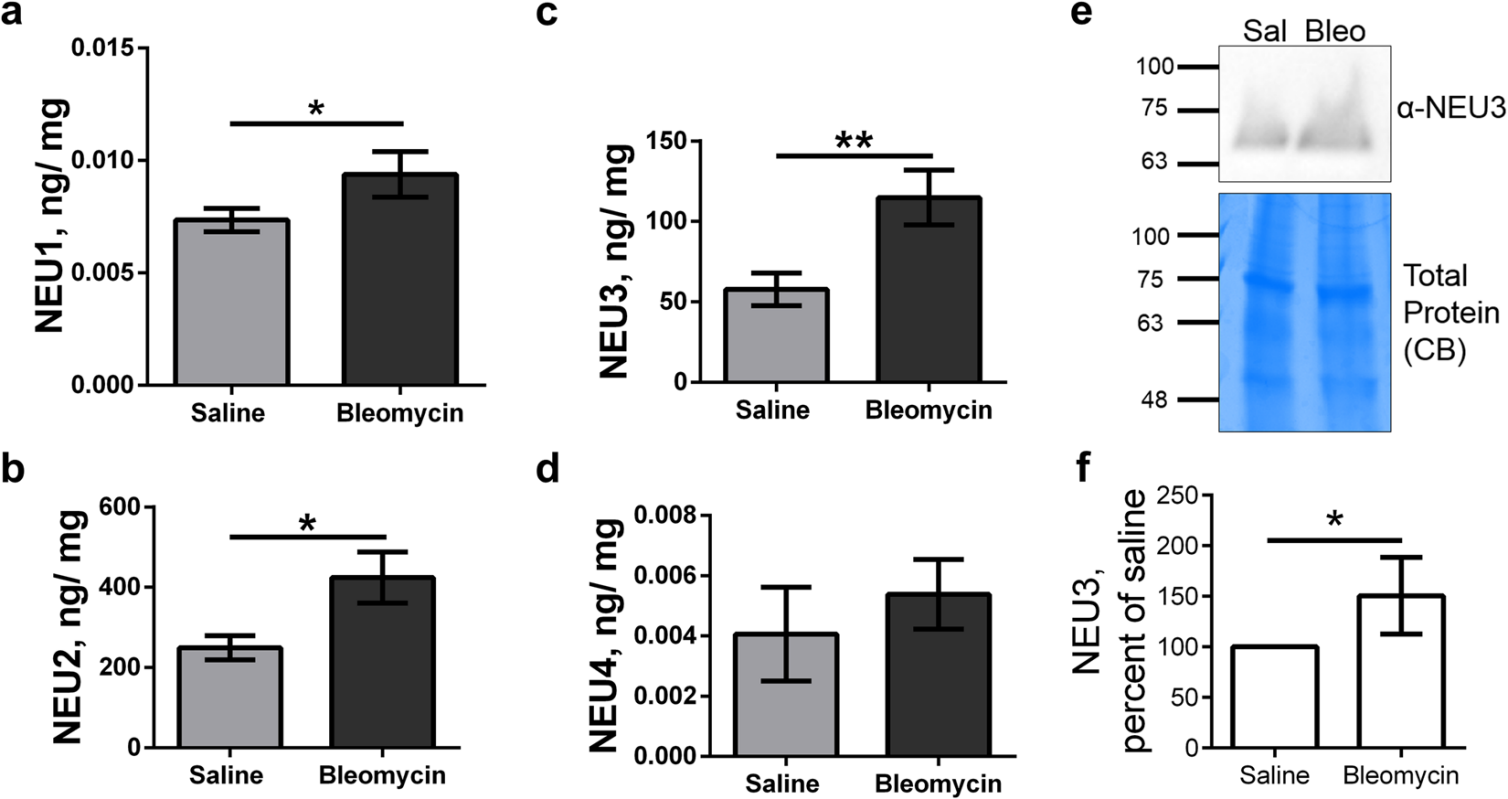
Detection of upregulated sialidases in bleomycin-treated mouse lung tissue lysates. Lysates from the indicated treatment groups were assayed for total protein and assayed by ELISA for NEU1 (**a**), NEU2 (**b**), NEU3 (**c**), NEU4 (**d**). (**e**) Western blot of lung tissue lysate from a saline (sal) and a bleomycin (bleo) treated mouse stained for NEU3 (upper panel); aliquots of the samples were run on a SDS-PAGE gel and stained with Coomassie brilliant blue (CB) (lower panel). The positions of molecular mass standards in kDa are at left. Images are representative of 3 pairs of mice. (**f**) Quantification of western blots. In (**a**–**f**) values are mean ± SEM, n = 3; *indicates p < 0.05 (t-test).

To further test the hypothesis that sialidases are upregulated in the fibrotic lungs, we prepared lung tissue lysates from saline and bleomycin treated mice. Total protein concentration and concentrations of the sialidases were measured in the lysates. Compared to saline controls, the bleomycin-treated mice had significantly higher levels of NEU1, NEU2 and NEU3, but not NEU4, in their lung lysates (Fig. 3a–d). In addition, on Western blots, compared to saline, bleomycin-treated mouse lung tissue lysates had significantly upregulated levels of NEU3 (Fig. 3e and f). Together, these results indicate that some sialidases are upregulated in pulmonary fibrosis in mice.

### The pro-fibrotic cytokine TGF-β1 upregulates sialidases in cultured cells

**Figure 4:**
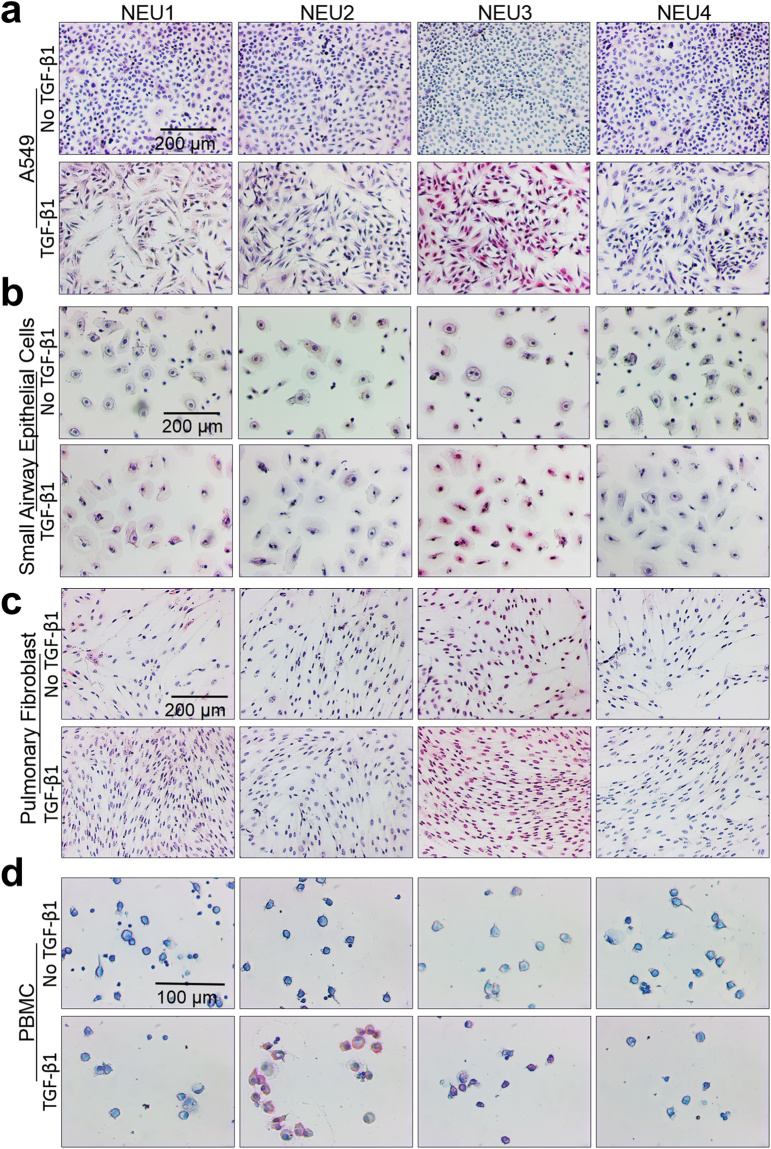
TGF-β1 increases sialidase expression in cultured cells. (**a**–**d**) The indicated human cell types were cultured in the presence or absence of 10 ng/ml TGF-β1 for 3 days and then stained for the indicated sialidases. All images are representative of 3 independent experiments. Bars are 0.2 mm in (**a**–**c**) and 0.1 mm in (**d**).

TGF-β1 is a key driver of fibrosis^1,61^. As the main cell types involved in lung fibrosis are epithelial cells, fibroblasts, and immune cells, we cultured the human alveolar basal epithelial adenocarcinoma cell line A549, human small airway epithelial cells, human pulmonary fibroblasts, and human immune cells (PBMC) with or without 10 ng/ml of recombinant active TGF-β1 (a standard concentration used in tissue culture experiments^6,62–64^) for five days. After five days, the cells were stained with antibodies against sialidases. TGF-β1 caused A549 cells to undergo a characteristic change in morphology^65^ and to increase levels of NEU3 and the percent of cells positive for NEU3 (Fig. 4a and supplementary Fig. S7a). TGF-β1 also caused human small airway epithelial cells to increase levels of NEU3, the percent of cells positive for NEU3 and slightly increased levels of NEU1 (Fig. 4b and supplementary Fig. S7b). As previously observed, TGF-β1 increased the proliferation of human pulmonary fibroblasts^66^, and caused these cells to increase levels of NEU3 (Fig. 4c and Supplementary Fig. S8). TGF-β1 increased levels of NEU2 and NEU3 in some cells in cultures of human PBMC (Fig. 4d and supplementary Fig. S7c).

### Sialidases show activity at neutral pH

The enzymatic activity of the four human sialidases has been characterized^67,68^, with >50% activity between pH of 3.5 to 5.5, and NEU2 and NEU4 are still active at neutral pH^67,69,70^. To determine if sialidases might have enzymatic activity at the pH of the extracellular environment, we assayed recombinant human sialidases at pH 6.4, approximately corresponding to the extracellular pH that might occur in a fibrotic tissue^71^, and at pH 7.0, approximately corresponding to a normal extracellular pH^71^. All four recombinant sialidases showed activity at pH 6.4 and pH 7.0 (Supplementary Table S2), indicating that sialidases could be active in an extracellular environment.

### NEU2 and NEU3 upregulate the intracellular and extracellular accumulation of TGF-β1 by PBMC

**Figure 5:**
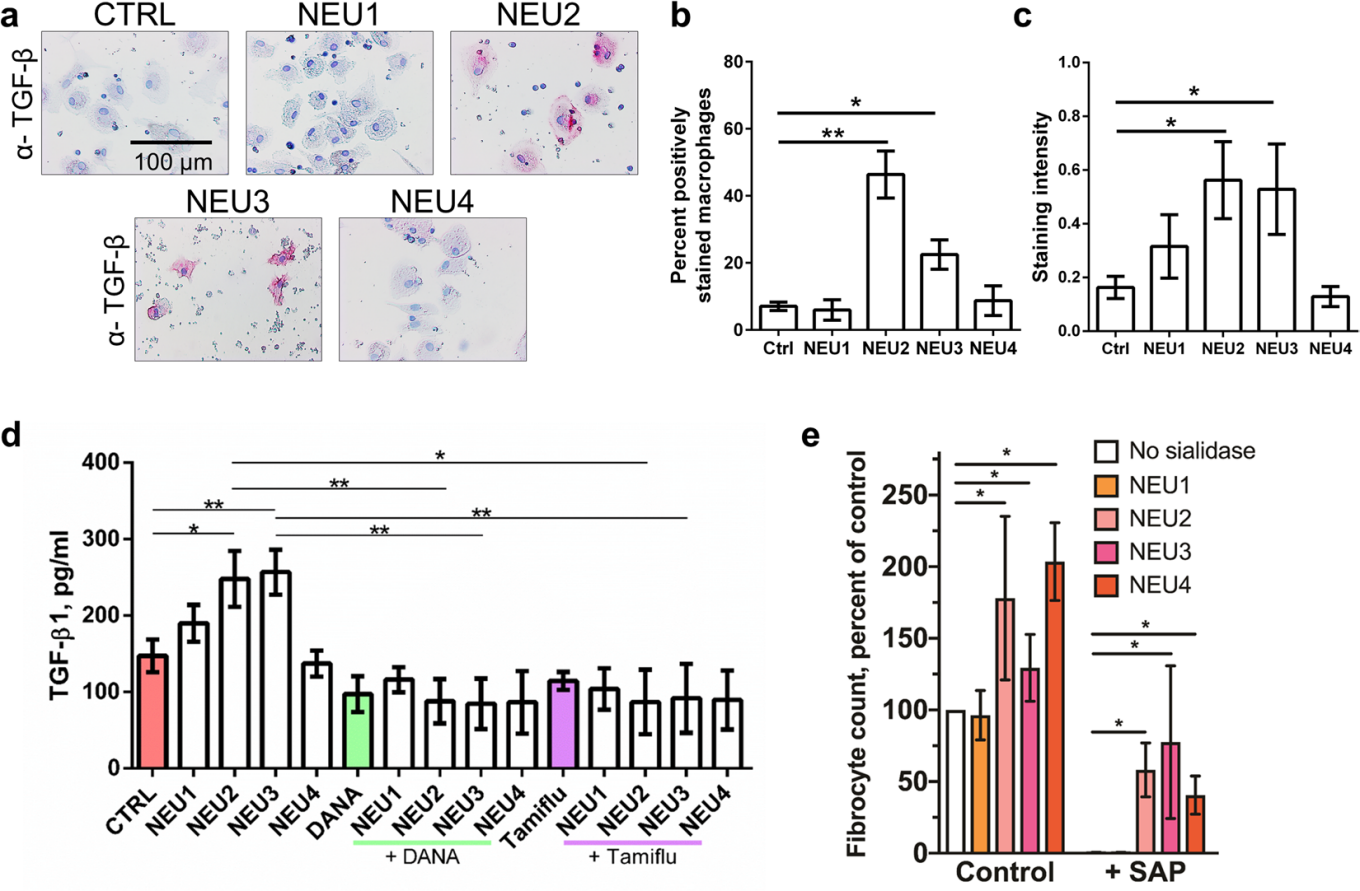
Some sialidases upregulate TGF-β1 and counteract the ability of SAP to inhibit fibrocyte differentiation. (**a**) Human PBMC were incubated with or without recombinant human sialidases for five days, then air dried and stained for TGF-β1. All images are representative of 3 independent experiments. Positive staining appears pink and counter staining is blue. Bar is 0.1 mm. (**b**) After staining, positively stained macrophages were counted and expressed as a percent of total cells. Values are mean ± SEM, n = 3. *p < 0.05, **p < 0.005 (t-test). (**c**) Quantification of staining intensity by ImageJ. Values are mean ± SEM, n = 3. *p < 0.05 (t-test). (**d**) Human PBMC were incubated with or without recombinant human sialidases, DANA, or Tamiflu for 5 days in serum-free medium. The conditioned media were analyzed by ELISA for total TGF-β1. Values are mean ± SEM, n = 7. *p < 0.05, **p < 0.01 (t tests). (**e**) Human PBMC were incubated in serum-free medium in the presence or absence of recombinant human sialidase or 2 µg/ml human SAP. After 5 days, fibrocytes were counted. Values are mean ± SEM, n = 3. *p < 0.05 (1-way ANOVA, Dunnett’s test). No fibrocytes were detected in the cultures with SAP and no sialidase or SAP and NEU1.

To determine if sialidases might cause cells to accumulate intracellular and extracellular TGF-β1, cells were cultured with sialidases. The levels of active TGF-β1 in or on the cells was detected by staining with an antibody against active TGF-β1 (Fig. 5a–c) and an ELISA was performed on the media supernatant to measure the extracellular accumulation of total TGF-β1 (Fig. 5d). When added to human PBMC, human NEU1 and NEU4 had no significant effect on the accumulation of TGF-β1, while NEU2 and NEU3 increased both cell-associated and extracellular TGF-β1 (Fig. 5a–d). The addition of the sialidase inhibitors DANA or Tamiflu blocked the effects of NEU2 and NEU3 on extracellular TGF-β1 (Fig. 5d), indicating that the effects of NEU2 and NEU3 are due to their sialidase activities. NEU 2 also caused some cells in PBMC to upregulate levels of NEU3 (Supplementary Fig. S9). These data suggest that sialidases might be able to potentiate fibrosis by increasing levels of extracellular TGF-β1.

### NEU2, NEU3, and NEU4 counteract the ability of SAP to inhibit fibrocyte differentiation

In support of the hypothesis that sialidases potentiate fibrosis, we observed that recombinant human NEU2, 3, and 4, when added to human PBMC, potentiate fibrocyte differentiation and counteract the ability of human SAP to inhibit fibrocyte differentiation (Fig. 5e). NEU1 however did not potentiate fibrocyte differentiation or counteract SAP.

### Sialidase inhibitors decrease fibrosis

**Figure 6:**
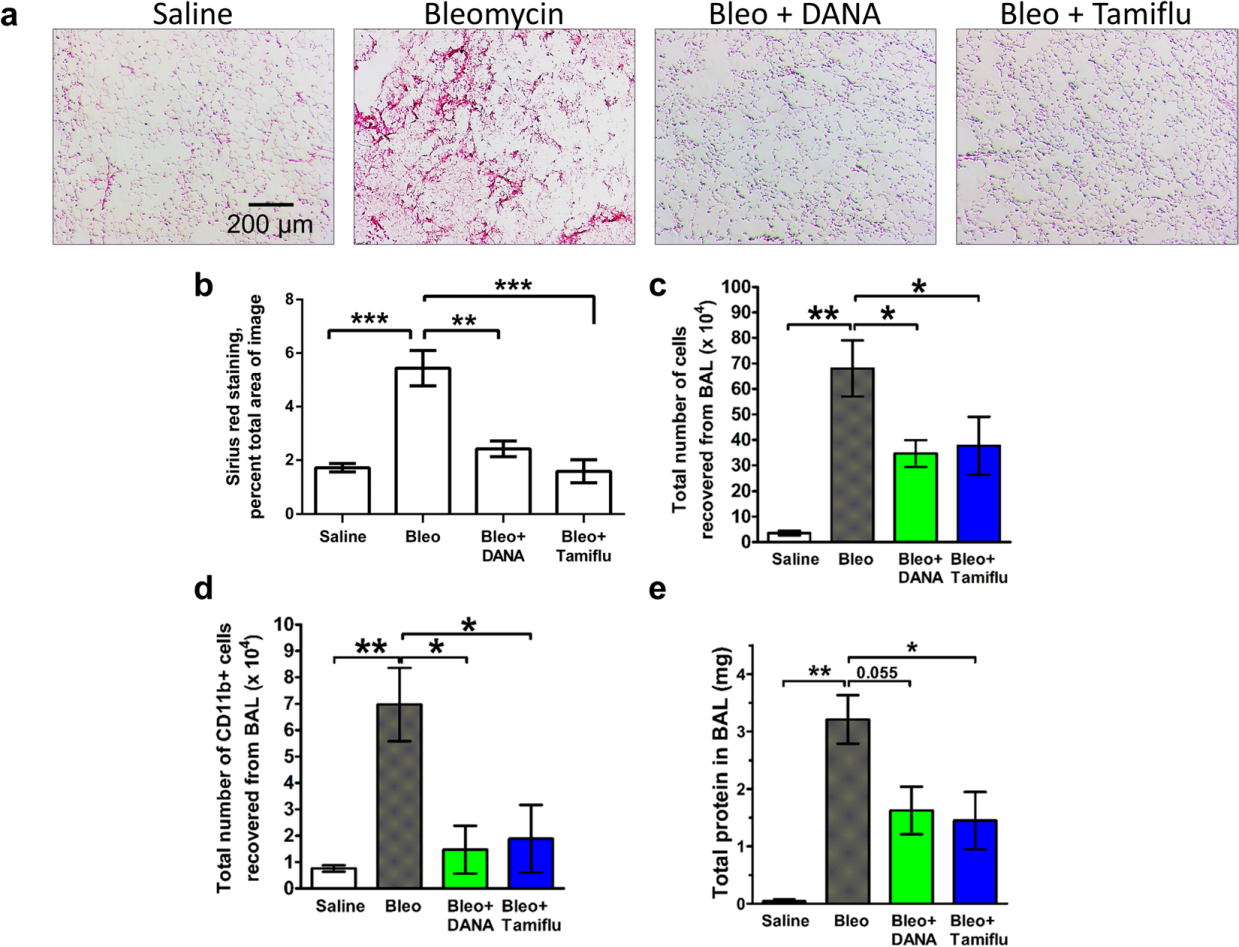
Inhibition of sialidases starting at day 10 after bleomycin attenuates fibrosis. (**a**) Sections of lung tissue from mice treated with bleomycin or saline, and then injected daily with saline, DANA, or Tamiflu starting at day 10 after bleomycin, and then euthanized at day 21, were stained for collagen with Sirus Red. Bar is 0.2 mm. All images are representative of 3 mice per group. (**b**) Quantification of staining with ImageJ. The percentage of area stained was quantified as a percentage of the total area of the lung. (**c,d**) The total number of cells and number of CD11b + cells in the BAL. (**e**) Total protein in the BAL. For B-E, values are mean ± SEM, n = 3 mice per group. *Indicates p < 0.05, **p < 0.01 (1-way ANOVA, Tukey’s test). In B and D, the differences between Saline, Bleo + DANA, and Bleo + Tamiflu were not significant.

**Figure 7:**
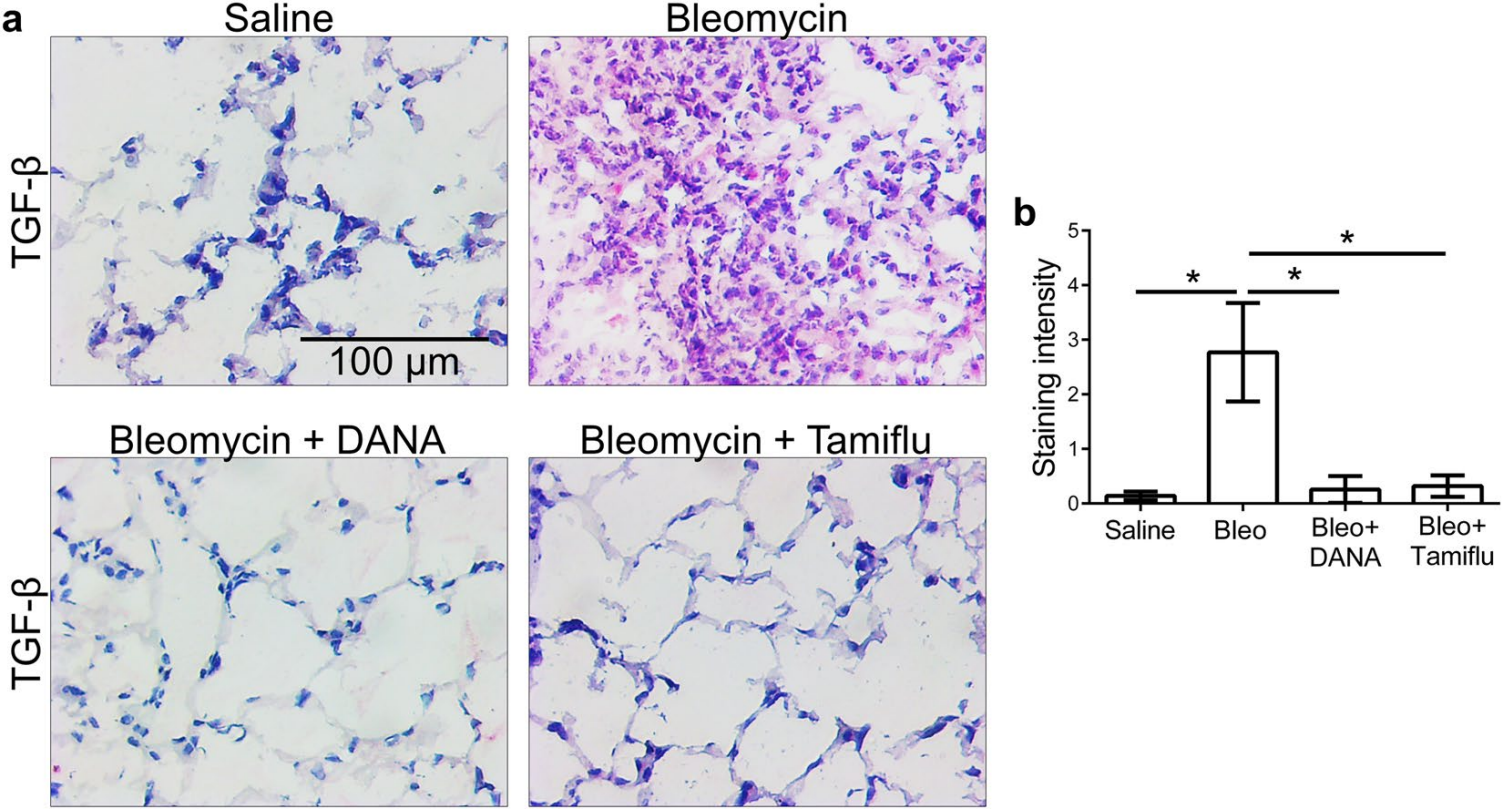
Sialidase inhibitors reduces TGF-β1 staining. (**a**) Sections of lung tissue from mice treated with bleomycin or saline, and then injected daily with saline, DANA, or Tamiflu starting at day 10 after bleomycin, and then euthanized at day 21, were stained for TGF-β1. All images are representative of 3 mice per group. (**b**) Quantification of staining intensity with ImageJ. Values are mean ± SEM, n = 3 mice per group. *Indicates p < 0.05 (t-test).

The sialidase inhibitor DANA inhibits all mammalian sialidases^72^. Although Tamiflu is a poor inhibitor of human sialidases, it is a potent inhibitor of murine sialidases, and inhibits LPS-induced mouse macrophage sialidase activity with an IC50 of 1 µm^40^. To test the hypothesis that blocking sialidase might decrease fibrosis, mice were treated with oropharyngeal bleomycin to induce symptoms of pulmonary fibrosis^60^ and then starting 10 days after bleomycin (when fibrosis has begun in this model^60^), mice were given daily intraperitoneal injections of 10 mg/kg DANA or 10 mg/kg Tamiflu. Both inhibitors are quite polar, and thus probably remain in the extracellular space. Assuming 2 ml of extracellular space in a 20 g mouse, these doses would then be ~3 µM. The DANA and Tamiflu treatments did not discernably affect mouse behavior or significantly affect mouse weights (Supplementary Fig. S10). At day 21, the DANA and Tamiflu treatments decreased the bleomycin-induced fibrosis, (Fig. 6a–c) and also counteracted the bleomycin-increased number of inflammatory CD11b cells in the BAL (Fig. 6d). Unlike SAP treatment^28^, Tamiflu treatment resulted in less protein in the BAL (Fig. 6e), suggesting that Tamiflu may inhibit edema or epithelial barrier destruction following bleomycin instillation. The DANA and Tamiflu treatments also reduced staining for TGF-β1 (Fig. 7). Similar results were observed with DANA and Tamiflu injections starting 24 hours after bleomycin, with the exception that DANA also significantly reduced BAL protein content (Supplementary Fig. S11). Together, these results suggest that sialidase inhibitors can decrease fibrosis.

In further support of the hypothesis that inhibiting sialidases can break a sialidase → fibrosis → sialidase positive feedback loop, we observed that DANA treatment starting 10 days after bleomycin decreased NEU1, NEU2 and NEU3 staining at 21 days (Supplementary Fig. S12). When staining of serial sections of fibrotic lesions with anti-NEU2 was done with DANA or Tamiflu present in the antibody and wash solutions, staining was still observed (Supplementary Fig. S10), indicating that residual DANA or Tamiflu that might have been present in the DANA or Tamiflu-treated lungs was not blocking the anti-NEU2 staining. Similar results were observed for DANA and Tamiflu not inhibiting NEU3 staining (Supplementary Fig. S13). Together, these results indicate that DANA and Tamiflu treatments reduce fibrosis, and appear to break the sialidase → fibrosis → sialidase positive feedback loop as evidenced by a reduction in sialidase levels.

## Discussion

In this report, we observe desialylation of glycoconjugates in human and mouse pulmonary fibrosis, which is likely due to the observed elevated levels of sialidases. We found what may be a positive feedback loop, with high levels of sialidases increasing extracellular TGF-β1 accumulation by human PBMC, and TGF-β1 increasing sialidases in human lung epithelial cells, fibroblasts, and PBMC. In support of the hypothesis that sialidases play a role in fibrosis, two different sialidase inhibitors strongly decreased fibrosis in the mouse bleomycin model of pulmonary fibrosis.

A recent report found increased levels of NEU1 in lung fibroblasts of some but not all pulmonary fibrosis patients^54^. We also observed increased NEU1 in mouse pulmonary fibrosis, and in one of three ILD patients. We also observed increased levels of NEU2 and NEU3 in mouse and pulmonary fibrosis, and observed that TGF-β1 can increase NEU2 and NEU3 in PBMC, and NEU3 in epithelial cells and fibroblasts. Other workers found elevated levels of sialidase activity in the BAL fluid from IPF patients^53^, and we detected upregulated levels of NEU3 in the BAL fluids from mice with bleomycin-induced pulmonary fibrosis. Compared to healthy individuals, in IPF patients there are no significant increases in the mRNAs encoding NEU1–4 ^73^. This suggests that, like many other proteins that are upregulated post-transcriptionally by TGF-β1^74^, sialidases may be upregulated post-transcriptionally by TGF-β1. In addition, sialidase protein production, secretion, and activity, can also be regulated by proteins that bind to sialidases, especially protective protein/cathepsin A (PPCA) binding to NEU1^16,19,75^. Together, these results suggest that post-transcriptional upregulation of multiple sialidases may contribute to fibrosis.

Desialylation of SAP blocks all of its known activities on cells^34^, and NEU2, 3, and 4 counteract SAP. These results suggest the possibility that in addition to sialidase upregulating TGF-β1 accumulation by immune cells, sialidase may potentiate fibrosis by desialylating SAP and inhibiting its activity, and that SAP is a key sentinel monitoring extracellular sialidase activity. The only known variation in serum SAP from healthy humans, other than concentration, is a small percentage of desialylated SAP^76–78^. Desialylated SAP is rapidly cleared from the circulation^77^, and an intriguing possibility is that the low serum SAP levels observed in patients with IPF^25,79^, myelofibrosis^80^, and non-alcoholic fatty liver disease^26^ may be due to increased sialidase activity associated with fibrosis. Some tumors have elevated levels of NEU3^68^. The high levels of NEU3 inhibit apoptosis, and intriguingly decreased apoptosis is a hallmark of fibrotic fibroblasts^81^. In tumors, high levels of NEU3 increase extracellular interleukin-6 (IL-6)^68^, and IL-6 is also associated with fibrosis^82^. Sialidases may thus potentiate fibrosis by a combination of increasing TGF-β1, causing clearance of SAP, inhibiting apotosis, and increasing IL-6.

Since fibrosis has very close similarities to the formation of scar tissue in a wound, a possible explanation for the fibrosis → sialidase → fibrosis positive feedback loop is that it may have originally evolved to accelerate wound healing. In electrical and control systems engineering, a small amount of positive feedback, as long as the gain of the system is less than 1 (i.e. a small amount of noise does not cause the system to oscillate or swing to one extreme or another), can dramatically increase both the sensitivity and the response time of a system^83^. If this feedback loop has a gain of less than 1, it would allow a faster response to wounds and faster wound healing. We think that in some individuals, or in some perturbed situations, the fibrosis → sialidase → fibrosis feedback loop develops a gain greater than 1, significantly contributing to fibrosis.

## Materials and Methods

### Cell isolation and culture

Human peripheral blood was collected from healthy volunteers who gave written consent and with specific approval from the Texas A&M University human subjects review board. All methods were performed in accordance with the relevant guidelines and regulations. Blood collection, isolation of PBMC, sources of culture media, supplements, and SAP, and PBMC culture and fibrocyte counts were done as described previously^84,85^ using 96 well plates (#89626 ibidi, Madison WI). Adenocarcinomic human alveolar basal epithelial cells (A549) (PromoCell, Heidelberg, Germany) were cultured in RPMI-1640 supplemented with 10% bovine calf serum (BCS) (Seradigm, Randor, PA), 100 U/ml penicillin, 100 µg/ml streptomycin, (Lonza, Walkersville, MD) and 2 mM glutamine (Invitrogen, Carlsbad, CA). Human small airway epithelial cells (Promocell) were cultured in small airway epithelial cell medium (PromoCell) following the manufacturer’s protocol. Human pulmonary fibroblasts (PromoCell) were cultured in DMEM supplemented with 10% BCS, 100 U/ml penicillin, 100 µg/ml streptomycin, and 2 mM glutamine. Where indicated, recombinant 10 ng/ml human TGF-β1 (Peprotech, Rocky Hill, NJ), 200 ng/ml recombinant human NEU1, 2, 3, or 4 (Origene, Rockville, MD), or 2 µg/ml human SAP (EMD Millipore, Billerica, MA; with the azide removed by centrifugal filters following^86^) were added to cells. Fibrocytes were counted as previously described^29,84^.

### Mouse model of pulmonary fibrosis

All procedures were done with specific approval of the Texas A&M University institutional animal care and use committee. All methods were performed in accordance with the relevant guidelines and regulations. Mice were treated with an oropharyngeal aspiration of 3 U/kg bleomycin (Calbiochem, Billerica, MA) in 0.9% saline or saline alone as previously described^28,34,87,88^. Starting either 10 days or 24 hours after bleomycin, when the bleomycin has been cleared from mice^89^, mice were given daily intraperitoneal injections of 10 mg/kg DANA (N-Acetyl-2,3-dehydro-2-deoxyneuraminic acid; EMD, Millipore) or oseltamivir (Tamiflu; Sigma, St. Louis, MO), both formulated as 4 mg/ml in PBS. Mice were euthanized 21 days after bleomycin aspiration, and bronchoalveolar lavage (BAL) cells collected, counted, and cytospins were prepared as described previously^28,34,87,88^. After BAL, lungs were inflated with and embedded in OCT compound (VWR), frozen, and stored at −80 °C. 6 μm sections on glass slides were air dried for 48 hours before use. BAL cytospins were prepared and immunochemistry were performed as described previously^28,34,87^ using anti- CD11b (clone M1/70 BioLegend, San Diego, CA) to detect blood and inflammatory macrophages or anti-CD11c (clone N418, BioLegend) to detect alveolar macrophages and dendritic cells with isotype-matched irrelevant antibodies (BioLegend) as controls. BAL fluid protein levels were measured as described previously^28^.

### Histology

HOPE-fixed chronic obstructive pulmonary disease (COPD) and idiopathic pulmonary fibrosis (IPF) patient lung sections were obtained from the Lung Tissue Research Consortium (NIH, Bethesda, MD). Slides were prepared for staining as described previously^85^ and incubated with 1 μg/ml biotinylated lectins SNA, PNA, and MAL II (Vector, Burlingame, CA) and then stained following the manufacturer’s instructions. Sections were also stained with 1 μg/ml of sheep polyclonal anti- ST3GAL2 (AF7275-SP, R&D Systems, Minneapolis, MN), anti- ST6GAL2 (AF7747-SP, R&D Systems) or anti- ST8SIA1 (AF6716-SP, R&D Systems), 1 μg/ml irrelevant sheep (#013-000-002, Jackson, West Grove, PA) or rabbit polyclonal antibody (#AB-105-C, R&D Systems, Minneapolis, MN), rabbit polyclonal anti-NEU1 (TA335236, Origene, Rockville, MD), anti-NEU2, (TA324482, Origene,) or anti-NEU4 (AP52856PU-N, Acris/Origene), in PBS/2% BSA (PBSB), or 0.5 μg/ml anti-NEU3 (TA590228, Origene) in PBSB/500 mM NaCl/0.1% NP-40 alternative (EMD Millipore, Billerica, MA) for 60 minutes. Washing 3 times with PBS for 10 minutes each and staining were then done as previously described^84,85^, except where required biotinylated donkey-anti-sheep (#713-066-147, Jackson) secondary antibody was used for staining. Where indicated, 1 mM DANA or 1 mM Tamiflu were added to the antibody incubation step and the subsequent wash steps. For PBMC, the medium was removed and the plate was air dried for 24 hours, treated with acetone for 20 minutes, dried for 10 minutes, hydrated with distilled water for 5 minutes, and then PBS for 5 minutes. For cells other than PBMC, the medium was removed and cells were fixed for 10 minutes with 2%(w/v) paraformaldehyde (EMS, Hatfield, PA) in PBS, blocked for 10 minutes in PBSB, and permeabilized for 8 minutes with PBS/0.1% (w/v) Triton X-100 (Alfa Aesar, Ward Hill, MA). Where indicated, PBMC, after five days incubation were air dried, then incubated with 1 µg/ml anti-active TGF-β1 antibodies (AB-100-NA, R&D Systems) in PBSB for 60 minutes. Staining of cells was then done as described above. After counter-staining, the plates were air-dried.

### Lung tissue lysate preparation

Approximately half lobes of lungs frozen in OCT were cut off, thawed, washed once in PBS, weighed, and then frozen in liquid nitrogen. 300 μl of Pierce RIPA buffer (Thermo Scientific, Rockford, IL) with 1x proteases and phosphatases inhibitor cocktail (Cell Signaling Technology, Danvers, MA) was added per 5 mg of tissue and the tissue was crushed with a pestle. The mixture was then incubated on a rotator for 2 hours at 4 °C. After centrifugation at 18000 × g for 10 minutes, the supernatant was collected as lung tissue lysate. Total protein was measured by OD 280/260 with a SynergyMX plate reader, (BioTek, Winooski, VT) using RIPA buffer with proteases/phosphatases inhibitor as a blank.

### Western blots

For Western blots, 20 μl of BAL fluid or lysate was mixed with 4 µl 5x Laemmli sample buffer and heated to 95 °C for 5 minutes. Western blots were done following^29^ with the exceptions that 4–20% Tris/glycine gels were from Lonza (EMD, Gibbstown, NJ). For analysis of lectin staining, western blots were pre-incubated with Carbo-Free Blocking Solution (Vector), and then incubated with biotinylated lectins diluted in the same carbo-free solution at 2 μg/ml for 30 minutes at room temperature. Labelling was detected with streptavidin-HRP (BioLegend), as described previously^28^. For other staining, blocking was in PBS/2% BSA/5% nonfat milk. Anti-NEU 1, 2, and 4 were incubated at 1:1000 in PBSB. For anti-NEU3, incubations were at 1:5000 in PBS/2% BSA/0.1% NP-40 alternative/0.01% SDS. All washes were in PBS/0.1% (v/v) Tween 20 (Fisher, Fair Lawn, NJ). SuperSignal West Pico Chemiluminescence Substrate (Thermo Scientific) was used following the manufacturer’s protocol to visualize the peroxidase using a ChemiDoc XRS + System (Bio-Rad, Hercules, CA).

### Sialic acid quantitation

Approximately 1.2 × 1.2 × 1.2 mm pieces from lungs frozen in OCT were thawed and washed 3 times in PBS and the lung piece was weighed. Sample hydrolysis and sialic acid measurement was done following^90^ using resorcinol (Alfa Aesar), with assays done in 900 µl final volumes. Sialic acid (Vector) was used for standards. Absorbances were measured with a SynergyMX plate reader. Values were then converted to mg sialic acid/g tissue.

### TGF-β1 ELISA

PBMC were cultured in 96 well plates at 5 × 10^5^ cells/ml and 200 μl/well in Fibrolife serum-free medium as described above. When the cells were plated, recombinant human sialidases were added to final concentrations of 200 ng/ml, and DANA or Tamiflu were added to 3 μM. After 5 days, culture supernatants were analyzed using a TGF-β1 ELISA kit, which detects total TGF-β1 (R&D Systems, Minneapolis, MN).

### Sialidase ELISAs

Lung tissue lysates were diluted to 100 µg total protein/ml in PBS. 55 µl of diluted lysate was added to a well of a 96-well Maxisorp immuno plate (#442404, Thermo Scientitic) and incubated at 4 °C overnight. Serial dilutions of recombinant NEU 1, 2, 3, and 4 (Origene) in PBS were also incubated and used for standard curves. The solutions were removed and the wells were blocked with 200 µl PBSB for 2 hours at room temperature with shaking. Anti- NEU1, 2, 3 and 4 antibodies (Origene) were then added in PBSB for 1 hour at room temperature following the manufacturer’s directions. After washing with PBSB, 1:1000 HRP-conjugated donkey-anti-rabbit IgG (Jackson) in PBSB was added for 1 hour. After washing, bound antibodies were detected using a TMB color development kit (Biolegend) and the reaction was stopped with 1 N HCl. Absorbances at 450 nm and 550 nm were measured using a SynergyMX plate reader.

### Image quantification

Images were converted to RGB stacks using Image J. The green channel (which shows the red staining) was used to adjust the intensity threshold level. The threshold level was kept the same for analyzing one set of images^91,92^. The area stained as a percentage of the total area of the image was then determined using Image J.

### Statistical Analysis

Data were analyzed by ANOVA, with appropriate post-tests, or t-test when appropriate, using Prism software (Graphpad, La Jolla, CA).

## Electronic supplementary material

Supplementary Information
