# Supplementary material for: Sialidase inhibitors attenuate pulmonary fibrosis in a mouse model
Source: Sci Rep. 2017 Nov 8;7:15069. doi: 10.1038/s41598-017-15198-8 (PMC5678159; doi:10.1038/s41598-017-15198-8)
Supplement: Supplementary file 1 — Supplementary Information [file 41598_2017_15198_MOESM1_ESM.docx]

Supplementary Data

**Sialidase inhibitors attenuate pulmonary fibrosis in a mouse model**

Tejas R. Karhadkar, Darrell Pilling, Nehemiah Cox, and Richard H. Gomer

Department of Biology, Texas A&M University,

301 Old Main Drive, College Station, Texas, 77843-3474 USA

Corresponding author:

Richard H. Gomer Ph.D.

Department of Biology

Texas A&M University

College Station, TX 77843-3474 USA

Phone: 979-458-5745

E-mail: rgomer@tamu.edu

**Materials and Methods**

*Sialidase activity assays*

Sialidase assays were done following ^1,2^ with the following modifications. PBS was adjusted to pH 6.4 or 7.0 with 12N HCl, and BSA was added to a final concentration of 100 µg/ml. Recombinant human NEU 1, 2, 3, or 4 were added to the PBS/ BSA to 300 ng/ml. 2′-(4-methylumbelliferyl)-α-D-N-acetylneuraminic acid sodium salt hydrate (4MU-NANA) (Sigma) was dissolved in water to 50 mg/ ml. After 30 minutes at room temperature, 4MU-NANA was added to the enzymes to 200 µM final concentration. 100 µl of the reaction was placed in the well of a 96-well plate and fluorescence was measured every 20 minutes at 37°C for 15 hours in a prewarmed SynergyMX plate reader with excitation at 360 nm and emission at 460 nm. The fluorescence in the absence of sialidases was subtracted from all readings. The fluorescence of known concentrations of 4-methylumbelliferone (Alfa Aesar) was used to convert fluorescence to moles of product.

*Flow cytometry*

Human pulmonary fibroblasts were cultured in the presence or absence of 10 ng/ml TGF-β1. After 3 days, the cells were detached using Accutase cell detachment solution (VWR) and were fixed for 10 minutes with 2% (w/v) paraformaldehyde (EMS) in PBS, followed by permeabilization for 10 minutes with 0.1% (w/v) Triton X-100 (Alfa Aesar) in PBS. Cells were then blocked for 10 minutes in PBSB. Cells were incubated for 60 minutes with 0.5 μg/ml anti-NEU3 or irrelevant rabbit antibody, as described above, the cells were washed twice with ice cold PBS. The cells were then incubated with 1:1000 goat anti-rabbit Alexa Fluor 647 (#A21245, Life Technologies, Carlsbad, CA) in PBS/ BSA and washed twice with PBS. The cells were then analyzed on an Accuri C6 flow cytometer (BD Bioscience) as previously described ^3^. The cells were kept on ice throughout the procedure.

*Human PMN isolation and culture, and leukemia cell culture*

Peripheral blood neutrophils (PMN) were isolated using Polymorphprep gradients (Axis-Shield, Oslo, Norway), as previously described ^4^. K562, U937, THP-1, NALM-6, and CHO-K1 cells (all from ATCC, Manassas, VA) and Mono Mac 6 (DSMZ, Braunschweig, Germany) were cultured in RPMI 1640 with 10% bovine calf serum (BCS) (VWR-Seradigm, Radnor, PA) containing 2 mM glutamine, 100 U/ml penicillin, and 100 μg/ml streptomycin (all from Lonza, Walkersville, MD). A549 cells, pulmonary fibroblasts, and PBMC were isolated and cultured as described in the main methods section. K562 is a chronic myeloid leukemia cell line ^5^, U937 is a lymphoma cell line ^6^, and THP-1 ^7^ and Mono Mac 6 ^8^ are acute myeloid leukemia cell lines. NALM-6 is a leukemic pre-B cell line ^9^. The CHO-K1 is an epithelial cell line derived from the ovary of the Chinese hamster ^10^. 2 x 10^6^ cells were lysed in RIPA buffer (#89900, Pierce, Rockford, IL) with 1X protease and phosphatase inhibitors (#5872S, Cell Signaling, Danvers, MA), on ice for 2 hours. The lysate was clarified by centrifugation at 18,000 x *g* for 10 minutes at 4˚C. The supernatant was collected and total protein concentration was estimated for each cell lysate. 10 µg of protein was loaded in each well. Western blotting with sialidase antibodies was as described in the main methods section. Coomassie staining was done as described previously ^11^.

*Specificity of sialidase antibodies*

800 ng of recombinant human NEU2 was added to 40 ng of anti-NEU2, or 800 ng of recombinant human NEU3 was added to 40 ng of anti-NEU3, in a total volume of 40 µl of PBSB. Protein:antibody mixtures were incubated at room temperature for 30 minutes, and then aggregates were removed by centrifugation at 18,000 x g for 5 minutes at 4 ^o^C. Human lung sections were stained as described in the main methods section with anti-NEU2 or anti-NEU3 antibodies with or without pre-incubation with recombinant protein.

**References:**

1 Potier, M., Mameli, L., Belisle, M., Dallaire, L. & Melancon, S. B. Fluorometric assay of neuraminidase with a sodium (4-methylumbelliferyl-alpha-D-N-acetylneuraminate) substrate. *Anal Biochem* **94**, 287-296 (1979).

2 Marathe, B. M., Lévêque, V., Klumpp, K., Webster, R. G. & Govorkova, E. A. Determination of Neuraminidase Kinetic Constants Using Whole Influenza Virus Preparations and Correction for Spectroscopic Interference by a Fluorogenic Substrate. *PLoS One* **8**, doi:10.1371/journal.pone.0071401 (2013).

3 Cox, N., Pilling, D., Gomer, R. H. DC-SIGN activation mediates the differential effects of SAP and CRP on the innate immune system and inhibits fibrosis in mice *Proceedings of the National Academy of Sciences* **112**, 8385-8390 (2015).

4 Herlihy, S. E., Pilling, D., Maharjan, A. S. & Gomer, R. H. Dipeptidyl Peptidase IV Is a Human and Murine Neutrophil Chemorepellent. *The Journal of Immunology* **190**, 6468-6477, doi:10.4049/jimmunol.1202583 (2013).

5 Klein, E. *et al.* Properties of the K562 cell line, derived from a patient with chronic myeloid leukemia. *International journal of cancer. Journal international du cancer* **18**, 421-431 (1976).

6 Sundstrom, C. & Nilsson, K. Establishment and characterization of a human histiocytic lymphoma cell line (U-937). *International journal of cancer. Journal international du cancer* **17**, 565-577 (1976).

7 Tsuchiya, S. *et al.* Establishment and characterization of a human acute monocytic leukemia cell line (THP-1). *International journal of cancer. Journal international du cancer* **26**, 171-176 (1980).

8 Ziegler-Heitbrock, H. W. *et al.* Establishment of a human cell line (Mono Mac 6) with characteristics of mature monocytes. *International journal of cancer. Journal international du cancer* **41**, 456-461 (1988).

9 Hurwitz, R. *et al.* Characterization of a leukemic cell line of the pre-B phenotype. *International journal of cancer. Journal international du cancer* **23**, 174-180 (1979).

10 Xu, X. *et al.* The genomic sequence of the Chinese hamster ovary (CHO)-K1 cell line. *Nat Biotechnol* **29**, 735-741, doi:10.1038/nbt.1932 (2011).

11 Pilling, D., Buckley, C. D., Salmon, M. & Gomer, R. H. Inhibition of fibrocyte differentiation by serum amyloid P. *Journal Of Immunology* **17**, 5537-5546 (2003).

12 Pilling, D., Fan, T., Huang, D., Kaul, B. & Gomer, R. H. Identification of markers that distinguish monocyte-derived fibrocytes from monocytes, macrophages, and fibroblasts. *PLoS ONE* **4**, e7475 (2009).

13 Luzina, I. G. *et al.* Elevated expression of NEU1 sialidase in idiopathic pulmonary fibrosis provokes pulmonary collagen deposition, lymphocytosis, and fibrosis. *Am J Physiol Lung Cell Mol Physiol* **310**, L940-954, doi:10.1152/ajplung.00346.2015 (2016).

**Supplementary figures**


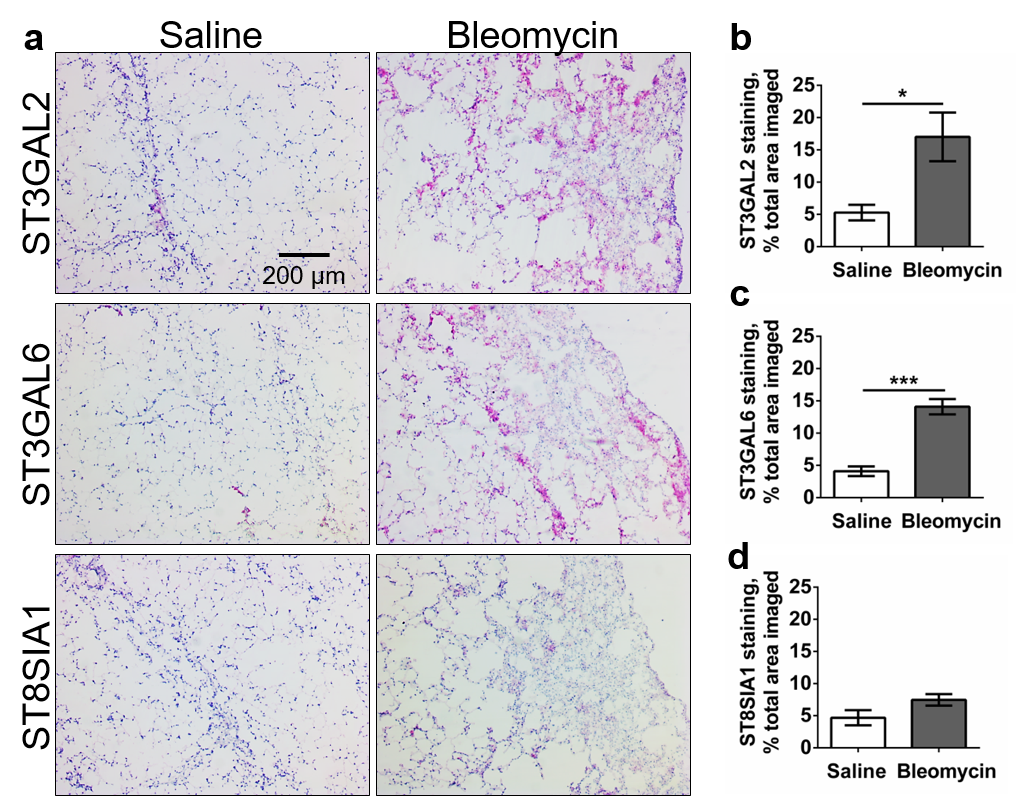


**Supplementary Figure S1. Sialyltrasferase expression in fibrotic mouse lungs. (a)** Sections of lungs from saline- or bleomycin- treated mice were stained with antibodies against sialyltransferase3 (ST3GAL2), sialyltransferase6 (ST3GAL6) and sialyltransferase8 (ST8SIA1). All images are representative of 3 mice per group. Bar is 0.2 mm. Quantification of staining for ST3GAL2 **(b)**, ST3GAL6 **(c)** and ST8SIA1 **(d)** with ImageJ. Values in B-D are mean ± SEM, n = 3 mice per group; * indicates p < 0.05, ***p < 0.001 (t-test).


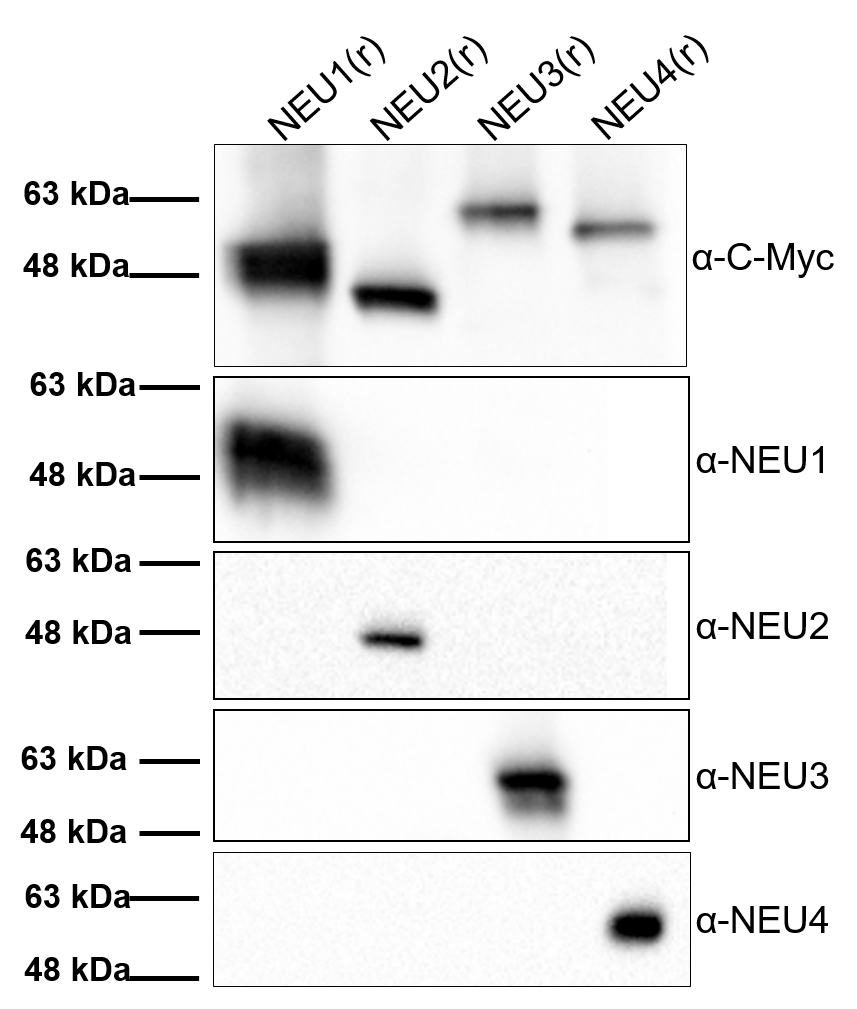


**Supplementary Figure S2. The specificity of anti-sialidase antibodies.** Western blots of recombinant (r) c-Myc tagged human sialidase proteins were stained with the indicated antibodies. Positions of molecular mass markers are at left. All four proteins were stained with anti-c-Myc antibodies (top panel). Each sialidase was detected by its corresponding antibody (bottom 4 panels). The antibody for a particular sialidase did not cross react with sialidases other than its target. All images are representative of three independent experiments.


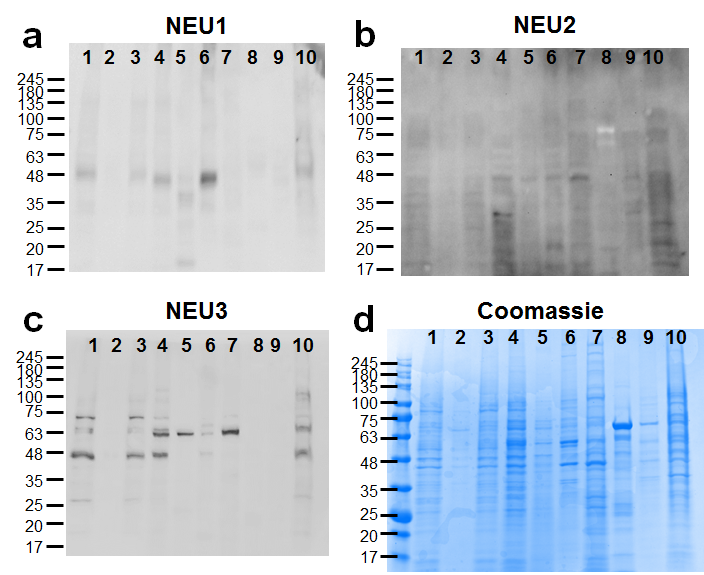


**Supplementary Figure S3.** S**ialidase expression in PBMC, PMN, and cell lines.** **a-c)** Western blots of cell lysates were stained with the indicated antibodies, and **d)** lysates were analyzed for total protein by Coomassie staining. Positions of molecular mass markers in kDa are at left. Lane 1 K562, lane 2 NALM-6, lane 3 THP-1, lane 4 Mono Mac 6, lane 5 U937, lane 6 human pulmonary fibroblasts, lane 7 ex vivo PBMC, lane 8 ex vivo PMN, lane 9 CHO-K1 cells, lane 10 A549 cells. Ex-vivo PBMC and PMN, along with Nalm6 and CHO cells, had no detectable staining with NEU1, whereas we detected a band of approximately 47 kDa in the other cell lysates. ForNEU2, we detected a band at ~44 kDa in the lysates from Mono Mac 6, U937, fibroblasts, and PBMC, but not the other cell lysates. For Neu3, we did not detect any bands in lysates from Naml-6, PMN, or CHO cells, but human lung fibroblasts, PBMC, and U937 cells had one band at ~65 kDa, and lysates from K562, THP-1, Mono Mac 6, and A549 cells had 3 bands at ~48, 65, and 75 kDa, as previously described ^13^.


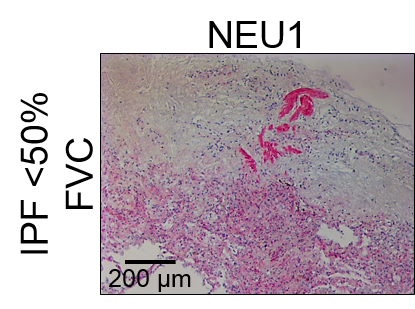


**Supplementary Figure S4. Upregulated NEU1 in one of three IPF patients.** Lung sections were stained as in Figure 2a. Bar is 0.2 mm.


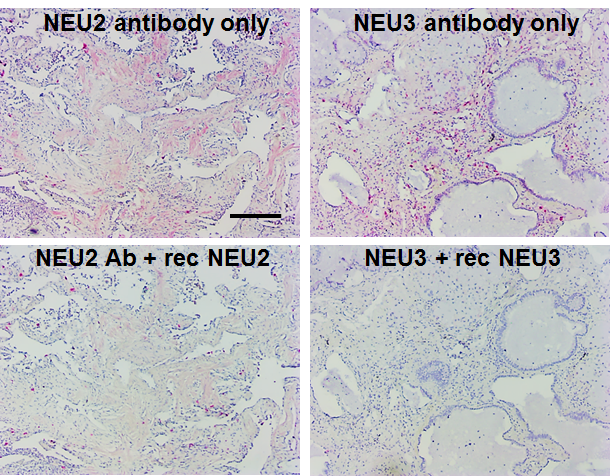


**Supplementary Figure S5. Specificity of sialidase antibodies.** To confirm the specificity of the NEU2 and NEU3antibodies, we pre-incubated the NEU2 and NEU3 antibodies with recombinant NEU2 or NEU3 respectively. Fibrotic human lung sections were then stained as described in Figure 2a. Bar is 0.2 mm. Pre-incubation of sialidase antibodies with recombinant protein abrogated staining, compared to sections incubated with sialidase antibodies alone.


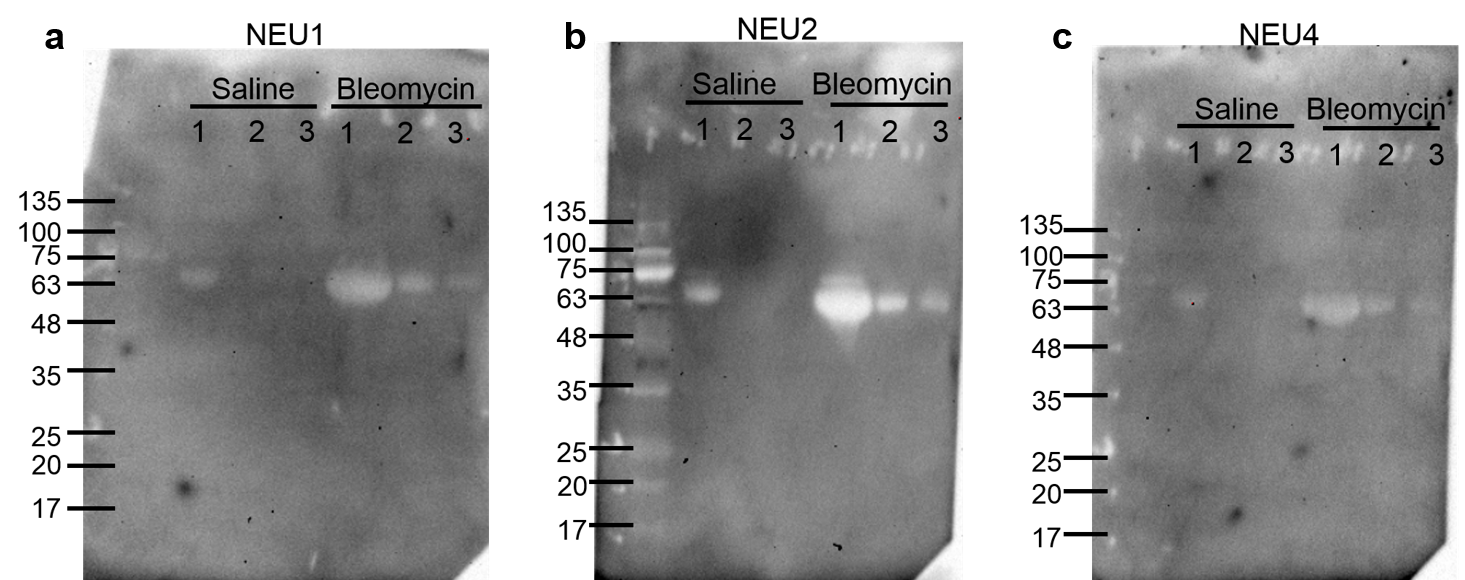


**Supplementary Figure S6:** **Sialidases 1, 2, and 4 are not detected in mouse BAL fluid.** BAL fluid from mice at day 21 after saline or bleomycin were stained for the indicated sialidases. 1, 2, and 3 refer to different individual mice. Positive staining would appear black. Molecular masses in kDa are at left. To determine if low levels of the sialidases were present, we over exposed the western blots. This reveal “negative” staining of albumin, especially in the BAL from bleomycin treated mice.


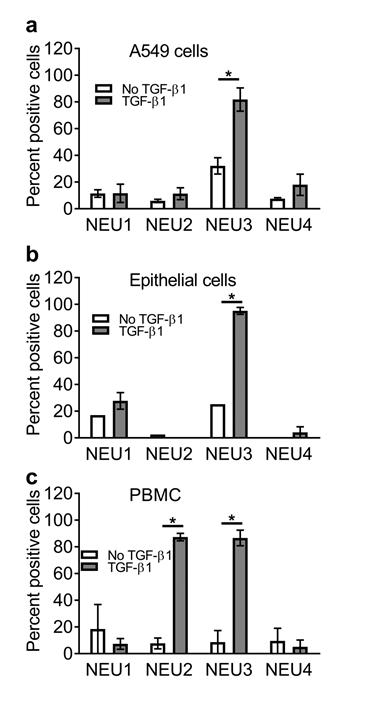


**Supplementary Figure S7. Quantification of TGF-β1 increased sialidase expression. a)** A549 cells, **b)** lung epithelial cells, and **c)** PBMC were incubated with and without TGF-β1 as in Figure 4c, and analyzed for sialidase expression. Values are mean ± SEM of the percent positive cells, n = 3. The absence of an error bar indicates that the error was smaller than the plot symbol. * p < 0.05 (t-test).


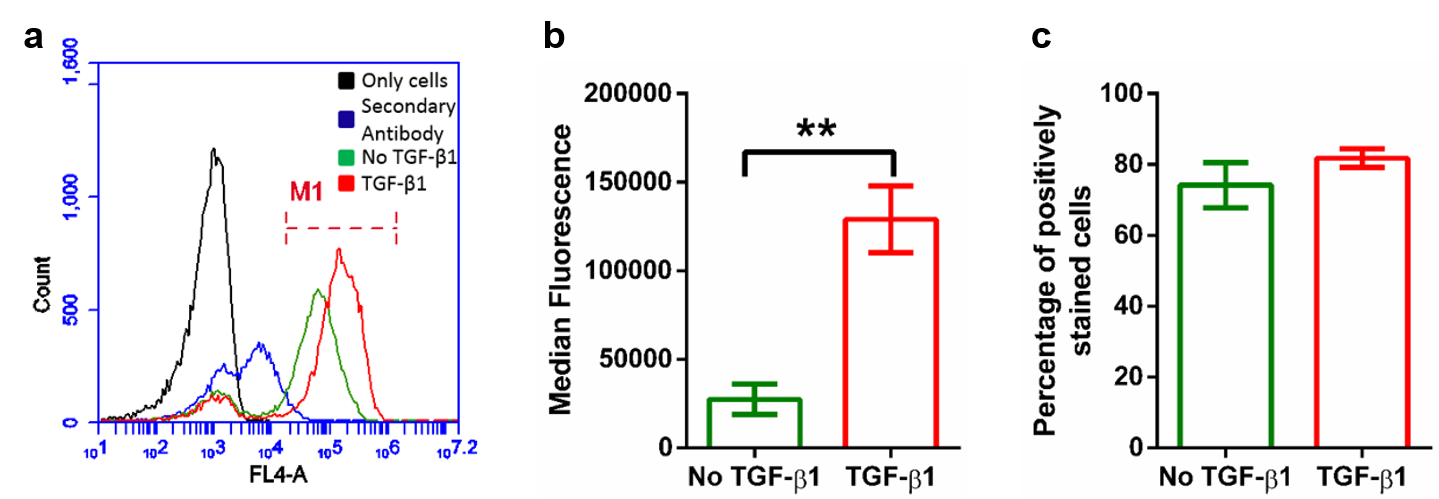


**Supplementary Figure S8. TGF-β1 increases NEU3 expression in human pulmonary fibroblasts.** Human pulmonary fibroblasts were incubated with and without TGF-β1 as in Figure 4c, and analyzed for anti- NEU3 staining by flow cytometry. **a)** Flow cytometry plot representative of 3 separate experiments. M1 indicates gate of positively stained cells used for panel **c**. **b)** Median fluorescence intensity in arbitrary units. **c)** Percentage of positively stained cells. Values in **b** and **c** are mean ± SEM, n = 3. ** p < 0.01 (t-test).


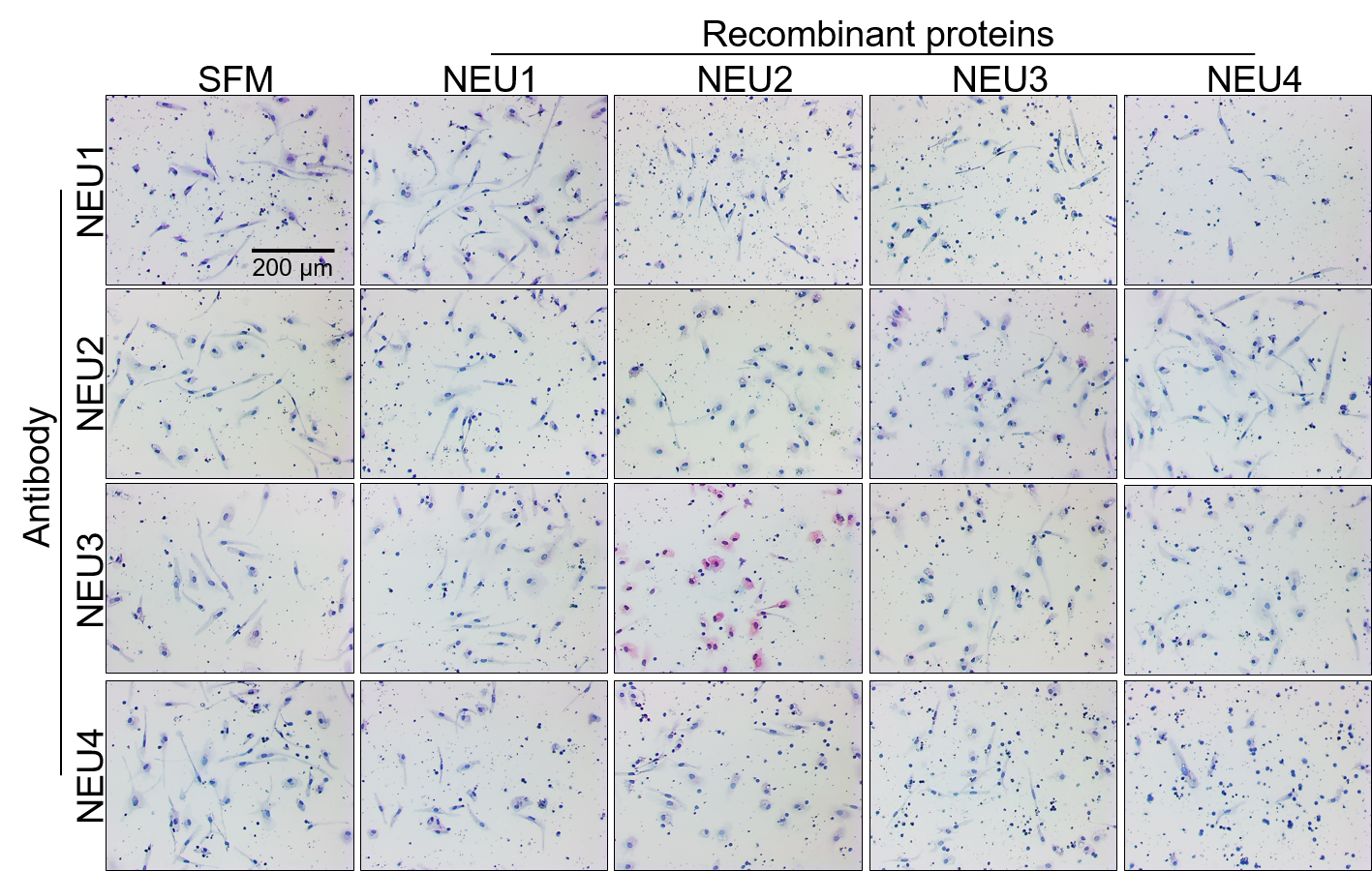


**Supplementary Figure S9. NEU2 causes increased expression of NEU3 in PBMC.** Human PBMC were cultured in serum free media (SFM) with or without recombinant human sialidases for 5 days, and then stained with antibodies against NEU1, NEU2, NEU3, or NEU4. Images are representative of 3 independent experiments. Bar is 0.2 mm.


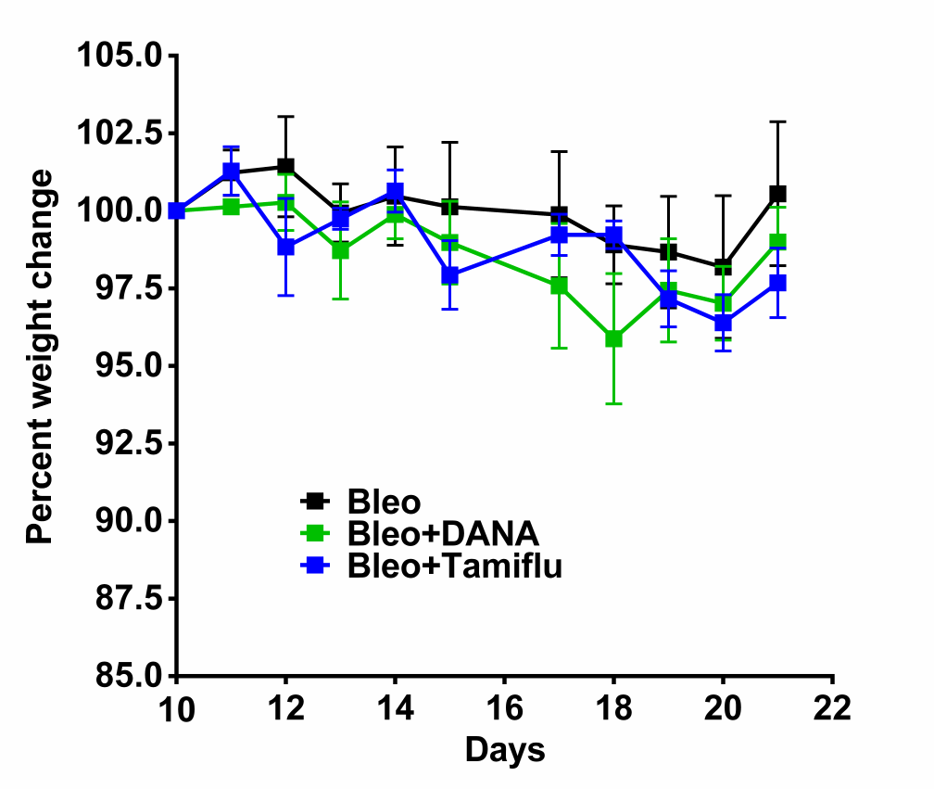


**Supplementary Figure S10. DANA and Tamiflu have no significant effect on the weight of bleomycin-treated mice.** Weights of mice used for the data in Figure 6 were recorded daily starting on day 10 (when mice were randomly assigned to the treatment groups). Values are mean ± SEM, n = 3 mice per group.


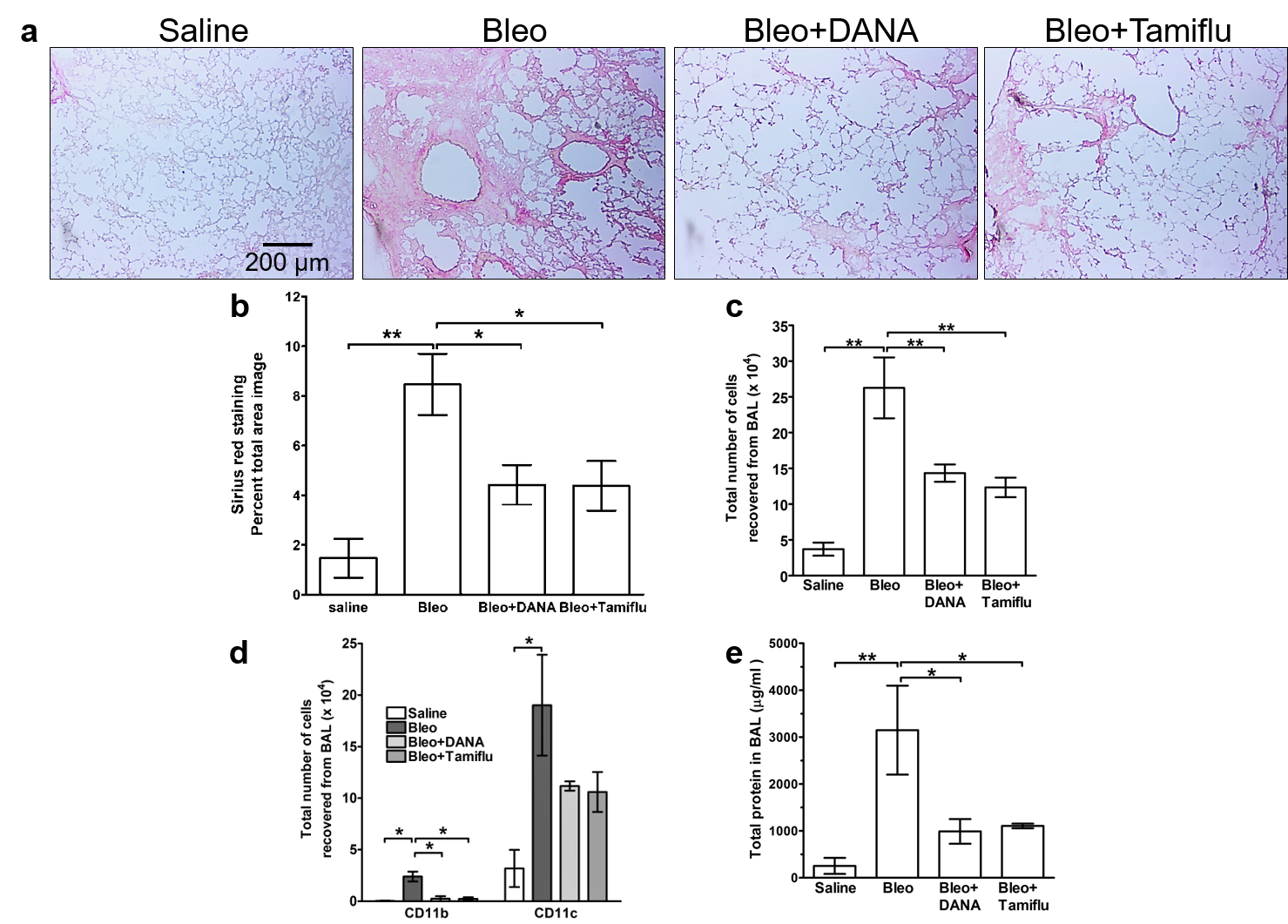


**Supplementary Figure S11. Inhibition of sialidases from day 1 attenuates fibrosis.** **a)** Sections of lung tissue from mice treated with bleomycin or saline, and then injected daily with saline, DANA, or Tamiflu starting at day 1 after bleomycin, and then euthanized at day 21, were stained for collagen with Sirius Red. Bar is 0.2 mm. All images are representative of 3 mice per group. **b)** Quantification of staining with ImageJ. The percentage of area stained was quantified as a percentage of the total area of the lung. **c, d)** The total number of cells, number of CD11b+ cells, and number of CD11c+ in the BAL. **e)** Total protein in the BAL. For B-E, values are mean ± SEM, n = 3 mice per group; * indicates p < 0.05, **p < 0.01 (1-way ANOVA, Tukey’s test).

**
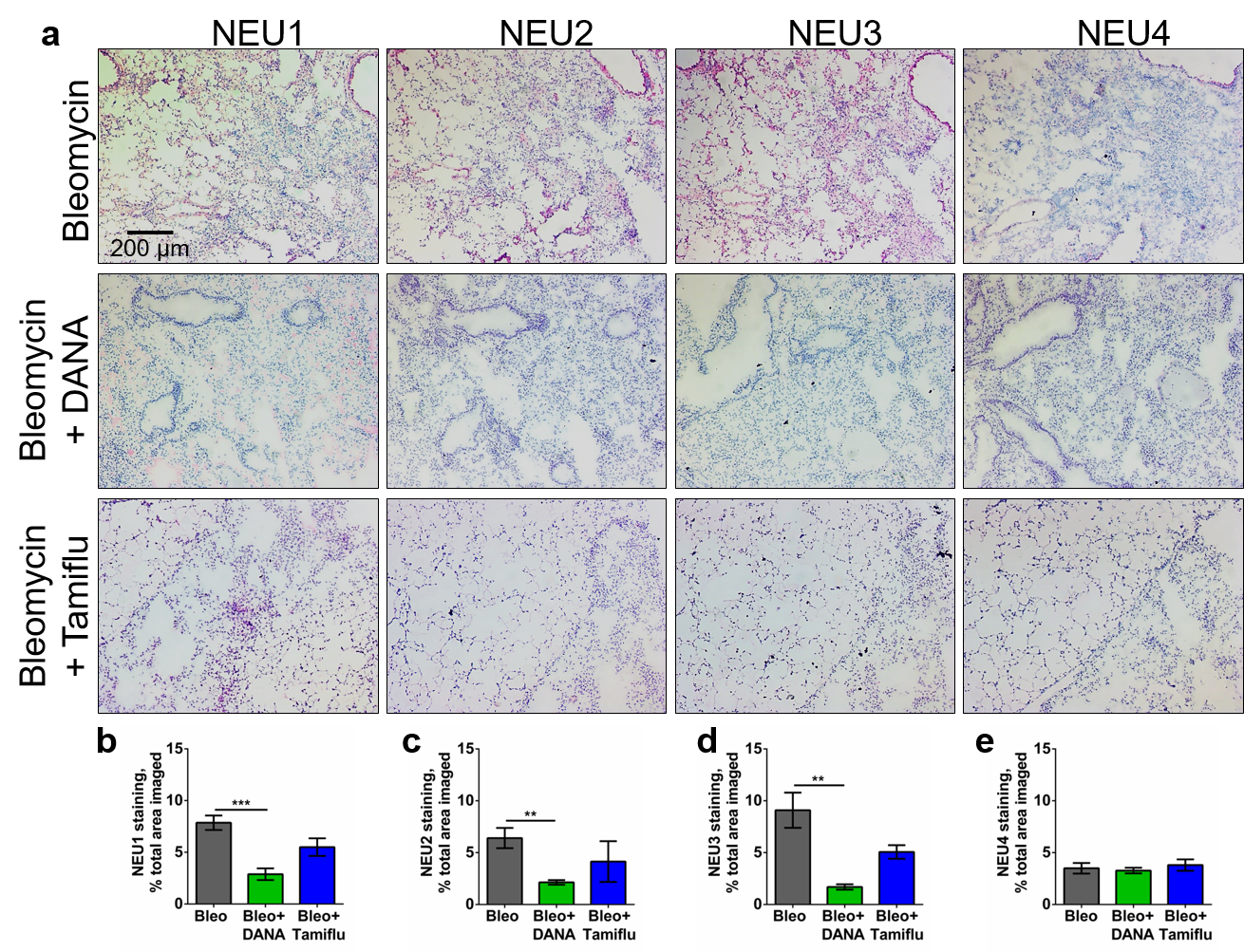
**

**Supplementary Figure S12. Sialidase inhibitors decrease NEU2 and NEU3 staining in bleomycin-induced lung fibrosis in mice.** **a)** Sections of lung tissue corresponding to Fig 6A were stained with antibodies against NEU1, NEU2, NEU3, and NEU4. Bar is 0.2 mm. All images are representative of 3 mice per group. Quantification of staining for NEU1 **(b)**, NEU2 **(c)**, NEU3 **(d)** and NEU4 **(e)** with ImageJ. Values are mean ± SEM, n = 3; ** indicates p < 0.005, ***p < 0.001 (t-test).

**
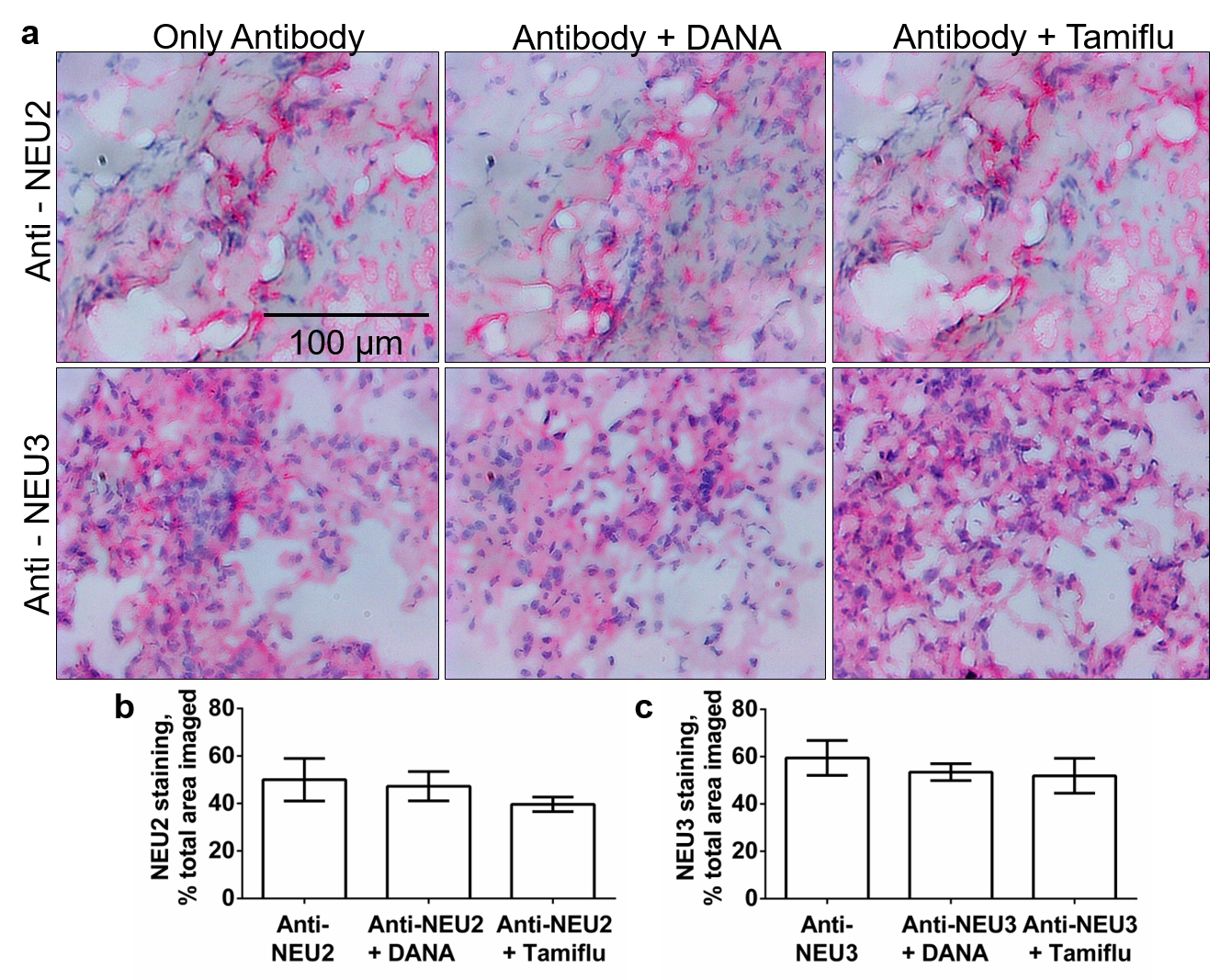
**

**Supplementary Figure S13. NEU2 and NEU3 staining are not blocked by residual DANA or Tamiflu.** **a)** Sections of lung tissue corresponding to the bleomycin treatment group in Figure 6 were stained with antibodies against NEU2 or NEU3 with or without 1 mM DANA or 1 mM Tamiflu present in the anti-NEU2 or anti-NEU3 antibody solution and the wash solutions between the NEU2 or NEU3 antibody incubation and the secondary antibody incubation. Bar is 100 µm. All images are representative of 3 mice per group. Quantification of images for NEU2 **(b)** and NEU3 **(c)** staining with ImageJ.

**Supplementary Table 1: Clinical details of human lung sections used in this study.**

| Group | FVC or FEV1 (mean ± SEM) | Gender | Age in years (mean ± SEM) | Clinical Details |
| --- | --- | --- | --- | --- |
| ILD <50% | 35.00 ± 3.396 | 3 males; 3 females | 46.00 ± 5.994 | n = 3 IPF /UIP,  n = 2 fibrosis,  n = 1 NSIP |
| COPD >80% | 83.80 ± 0.6633 | 3 males; 2 females | 64.80 ± 5.417 | n = 3 COPD,  n = 2 emphysema |
| t-test | p < 0.0001 | ns | p = 0.0483 |  |

**S1 Table: Clinical details of the human lung sections used in the study.** FVC indicates forced vital capacity; COPD indicates chronic obstructive pulmonary disease; ILD indicates interstitial lung disease; Fibrosis indicates uncharacterized ILD; UIP indicates usual interstitial pneumonia; IPF indicates idiopathic pulmonary fibrosis; NSIP indicates non-specific interstitial pneumonia. Because of a limited number of lung tissue sections obtained from the LTRC, each type of staining was performed on a randomly selected set of 4 of the 5 patients from each group.

**Supplementary Table 2: Sialidase activity at neutral pH**

| Activity, μmole / min / mg protein | | |
| --- | --- | --- |
| Sialidase | pH 6.4 | pH 7.0 |
| NEU1 | 12.8 ± 1.3 | 7.2 ± 0.9 |
| NEU2 | 16.4 ± 1.2 | 8.9 ± 1.2 |
| NEU3 | 15.2 ± 1.0 | 8.3 ± 1.2 |
| NEU4 | 3.8 ± 0.8 | 2.6 ± 0.9 |

**S2 Table: Sialidases have activity at neutral pH.** Recombinant human sialidases were assayed for activity at pH 6.4 and pH 7.0. Values are mean ± SEM, n=3.
